# Exogenous pyruvate represses histone gene expression and inhibits cancer cell proliferation via the NAMPT–NAD^+^–SIRT1 pathway

**DOI:** 10.1093/nar/gkz864

**Published:** 2019-10-10

**Authors:** Rui Ma, Yinsheng Wu, Yansheng Zhai, Bicheng Hu, Wei Ma, Wenqiang Yang, Qi Yu, Zhen Chen, Jerry L Workman, Xilan Yu, Shanshan Li

**Affiliations:** 1 State Key Laboratory of Biocatalysis and Enzyme Engineering, School of Life Sciences, Hubei University, Wuhan, Hubei 430062, China; 2 The Central Laboratory, Wuhan No.1 Hospital, Wuhan, Hubei 430022, China; 3 Hubei Cancer Hospital, Tongji Medical College, Huazhong University of Science and Technology, Wuhan, Hubei 430079, China; 4 Stowers Institute for Medical Research, 1000 E. 50th Street, Kansas City, MO 64110, USA

## Abstract

Pyruvate is a glycolytic metabolite used for energy production and macromolecule biosynthesis. However, little is known about its functions in tumorigenesis. Here, we report that exogenous pyruvate inhibits the proliferation of different types of cancer cells. This inhibitory effect of pyruvate on cell growth is primarily attributed to its function as a signal molecule to repress histone gene expression, which leads to less compact chromatin and misregulation of genome-wide gene expression. Pyruvate represses histone gene expression by inducing the expression of NAD^+^ biosynthesis enzyme, nicotinamide phosphoribosyltransferase (NAMPT) via myocyte enhancer factor 2C (MEF2C), which then increases NAD^+^ levels and activates the histone deacetylase activity of SIRT1. Chromatin immunoprecipitation analysis indicates that pyruvate enhances SIRT1 binding at histone gene promoters where it reduces histone acetylation. Although pyruvate delays cell entry into S phase, pyruvate represses histone gene expression independent of cell cycle progression. Moreover, we find that administration of pyruvate reduces histone expression and retards tumor growth in xenograft mice without significant side effects. Using tissues from cervical and lung cancer patients, we find intracellular pyruvate concentrations inversely correlate with histone protein levels. Together, we uncover a previously unknown function of pyruvate in regulating histone gene expression and cancer cell proliferation.

## INTRODUCTION

Cancer cells reprogram their metabolism to support their demands for rapid growth and proliferation (1). This metabolic reprogramming is a hallmark of many types of cancer and the prominent rewired metabolism involves elevated glucose uptake and accelerated glycolysis flux to lactate even in the presence of functional mitochondria and sufficient oxygen. This phenomenon is known as the ‘Warburg effect’ or aerobic glycolysis (2,3). This metabolic reprogramming provides cancer cells with ATP and biosynthetic building blocks, including intermediate metabolites, biosynthesis of nucleotides, proteins and membrane components (4). As cancer cells rely heavily on aerobic glycolysis for survival and proliferation (3), decoding the precise function of glycolytic enzymes and metabolites in carcinogenesis and identifying the crucial nodes that differentiate pathological and healthy cell behavior will provide insights into the development of novel predictive biomarkers and anti-cancer drugs (5,6).

Many glycolytic enzymes and metabolites have been reported to regulate histone modifications and gene expression (7). Some metabolites serve as essential cofactors or substrates for chromatin-modifying enzymes to modify histones and control the transcription process (4,8). For example, ∼5% glucose is needed for hexosamine biosynthetic pathways to produce *N*-acetylglucosamine (GlcNAc), which is the donor for histone glycosylation (9). Glucose can also be metabolized to acetyl-CoA, which in turn regulates histone acetylation and chromatin structure (10,11). Lactate has been reported to function as a weak inhibitor of histone deacetylases (HDACs) to increase gene transcription (12). Some glycolytic enzymes such as pyruvate kinase PKM2 and hexokinase (HK1) have been reported to function as protein kinases that phosphorylate histones, establishing a direct link between metabolism and histone modifications (13,14). We have previously reported that pyruvate kinase forms a complex, SESAME to phosphorylate histone H3T11 and regulate gene expression (15). Moreover, glycolysis is required for SESAME to phosphorylate H3T11 by supplying phosphoenolpyruvate (PEP) as the substrate and fructose 1,6-biphosphate (FBP) as the coactivator (16). However, for most glycolysis metabolic enzymes and metabolites, little is known about their roles in histone modifications and gene expression.

Pyruvate is the end-product of glycolysis. It is a key intersection in multiple metabolic pathways required for ATP production as well as homeostasis of carbohydrates, fats and amino acids (17,18). It has been proposed that pyruvate is able to antagonize oxidative stress by reacting with hydrogen peroxide (19). Due to its anti-oxidative properties, pyruvate has a protective role in renal damage, ischemia, epilepsy and hypoxia in non-malignant tissues (20–22). However, little is known about the impact of pyruvate on cancer cell growth and gene expression. In this study, we investigated the effect of exogenous pyruvate on the proliferation of a panel of cancer cells and found that it significantly inhibits cell growth. Moreover, our data showed that pyruvate treatment inhibits cell proliferation primarily by repressing histone gene expression. We also uncovered the underlying mechanism by which pyruvate represses histone gene expression, providing a novel signaling pathway that connects cell metabolism with histone gene expression and tumorigenesis.

## MATERIALS AND METHODS

### Materials

The siRNA sequences used to target each protein were listed in Supplementary Table S1. The primers used for ChIP and qRT-PCR were listed in Supplementary Table S2. All antibodies and other critical reagents used in this study are described in Supplementary Table S3.

### Cell culture

The HeLa, SiHa, HepG2, MCF-7 and HCC cells were obtained from the American Type Culture Collection (ATCC). The MDA-MB-231 cells are provided by Dr Peijing Zhang from Huazhong University of Science and Technology. The cell lines involved in our experiments were reauthenticated by short tandem repeat analysis after resuscitation in our laboratory. The HeLa, SiHa, HepG2, HCC and MDA-MB-231 cells were maintained in Dulbecco's modified Eagle's medium (DMEM) supplemented with 10% fetal bovine serum and 1% of penicillin/streptomycin solution. MCF-7 cells were maintained Eagle's minimum essential medium (EMEM) supplemented with 10% fetal bovine serum and 1% of penicillin/streptomycin solution. For pyruvate treatment, cells were grown to 40% confluency and 5 mM sodium pyruvate was added to medium in most cases. As a control, cells were treated with the same concentration of NaCl in parallel.

### Vector construction

Plasmids pCMV-H3 and pCMV-H4 were purchased from Genecreate Inc. (Wuhan, China). Plasmids pCMV-H2A, pCMV-H2B, pCMV-MEF2C, pCMV-NAMPT and pCMV-SIRT1 were constructed by inserting the corresponding gene fragments into pCMV-control (Genecreate Inc.). The target gene fragments were amplified from the cDNA of HeLa cells using the primers listed in Supplementary Table S2.

To construct the shSIRT1 vector, a 21-mer short hairpin RNA (shRNA) against SIRT1 mRNA (GeneBank no. AF083106.2) was designed. The shRNA sequence contains a 9-bp loop sequence that separates the two complementary domains. The following are sequences for complete SIRT1 shRNA insert: 5′-GATCCGCTTGATGGTAATCAGTATCTTTCAAGAGAAGATACTGATTACCATCAAGCTTTTTTGGAAA-3′ (sense) and 5′-AGCTTTTCCAAAAAAGCTTGATGGTAATCAGTATCTTCTCTTGAAAGATACTGATTACCATCAAGCG-3′ (antisense). These oligonucleotides were cloned into the pSilencer 2.1 shRNA vector (Invitrogen) to generate pSIRT1 shRNA. All plasmids were verified by DNA sequencing.

### RNA interference (RNAi)

The siRNA sequences used in this study were listed in Supplementary Table S1. Cells were transfected with siRNA using Lipofectamine 3000 according to the manufacturer's instructions (Invitrogen). Stable knockdown cells were prepared by transfecting with corresponding shRNAs and selected with puromycin. The knockdown efficiency was determined by qRT-PCR and/or western blots.

### Western blots analysis

Western blots analysis was performed on whole cell lysate as described previously (23). Protein samples were separated by 8–15% SDS-PAGE and transferred to Immobilon-P PVDF membrane. The blots were probed with antibodies listed in Supplementary Table S3.

### MNase digestion assay

MNase digestion assay was performed as described previously (23). Cells were permeabilized with 0.025% lysolecithin (Sigma) in permeabilization solution (150 mM sucrose, 80 mM KCl, 35 mM HEPES, pH7.4, 5 mM K_2_HPO_4_, 5 mM MgCl_2_, 0.5 mM CaCl_2_) for 2 min. The cells were then digested with indicated amounts of MNase (0–100 U) in digestion buffer (150 mM sucrose, 50 mM Tris, pH 7.5, 50 mM NaCl, 2 mM CaCl_2_) for 8 min. The reaction was stopped with lysis buffer (20 mM Tris, pH 7.4, 200 mM NaCl, 2 mM EDTA, 2% SDS, 0.2 mg/ml proteinase K, 0.2 mg/ml RNase A). DNA was purified by phenol–chloroform extraction and ethanol precipitation. Five hundred nanogram DNA was loaded in agarose gel and stained with ethidium bromide.

### RNA sequencing (RNA-seq) by Illumina HiSeq

Total RNA was extracted from cells using TRIzol (Invitrogen) and the quality was examined using Agilent 2100 Bioanalyzer according to the manufacturer's instructions. Library construction, sequencing and bioinformatics analysis were done by GENEWIZ Inc. (Suzhou, China). There are three biological replicates for control and pyruvate treatment. Differential expression levels of aligned sequences were calculated using significant thresholds set at fold change over two and adjusted *P* value ≤0.05.

### Quantitative real time RT-PCR (qRT-PCR)

Total RNA was extracted from cells using RNAiso Plus (Takara) and treated with DNase I (RNase-free) (Takara) according to manufacturer's instructions. 0.5 μg total RNA was reverse transcribed to cDNA using Reverse Transcriptase Kit (M-MLV) (ZOMANBIO). The cDNA was diluted 1:10 prior to PCR amplification and then subjected to real time PCR in a Bio-Rad CFX96 Real-Time PCR Detection System using SYBR Green PCR Master Mix (Bio-Rad) as described previously (24). The primers used for qRT-PCR were listed in Supplementary Table S2. The relative mRNA levels were determined by the ΔΔCt quantification method using the CFX manager 3.1 (Bio-Rad). Actin mRNA levels were used as internal controls. The validity of the qRT-qPCR data was assured by following the MIQE guidelines (25).

### Cell proliferation and cell cycle analysis

Cells were cultured in 96-well plates and treated with 0–50 mM sodium pyruvate. After 24 h, the cell proliferation rate was determined by the Cell Counting Kit (CCK-8, Dojindo, Japan) according to the manufacturer's instructions. Briefly, 2  ×  10^3^ cells/well were seeded in 96-well culture plates and treated with different concentrations of sodium pyruvate. CCK-8 solution was then added and the absorbance at 450 nm was measured. To avoid the osmotic stress caused by Na^+^, cells were treated with either 5 mM sodium pyruvate or 5 mM NaCl. Cell numbers were then counted at different time points.

For Colony formation assay, cells were plated into a six-well tissue culture plate (∼500 cells/well) at 37°C. The resulting colonies were fixed with methanol for 10 min, stained with methylthionine chloride and photographed.

For cell cycle analysis, cells were first synchronized with 1.5 mM hydroxyurea (HU). Cells were then washed twice in PBS and grown in fresh medium with or without sodium pyruvate. Cells were collected at different time points and fixed with 70% ethanol overnight. Cells were then stained with 50 μg/ml propidium iodide (PI) and measured by Flow cytometry (Beckman coulter, CytoFLEX) as described previously (26). The data were analyzed with Modfit LT 4.1 according to the manufacturer's instructions.

### Apoptosis assays

HeLa cells were treated with 5 mM sodium pyruvate or 5 mM NaCl for 24 h. Cells were then subjected to flow cytometry analysis using Annexin V-FITC/PI according to the manufacturer's instructions.

### Chromatin immunoprecipitation (ChIP) assay

ChIP experiments were performed as described previously (15). Cells were cross-linked with 1% formaldehyde and quenched by 0.125 M glycine. Cells were collected, washed and lysed in lysis buffer. DNA was sheared by sonication and subjected to immunoprecipitation with antibodies pre-bound to Protein G Dynabeads overnight. Beads were washed and the eluted DNA/protein complexes were treated with 20 μg Proteinase K at 55°C for 2 h and reverse crosslink at 65°C overnight. The purified RNase A digested DNA were quantitated by qPCR with specific primers listed in Supplementary Table S2.

### Quantitation of intracellular NAD^+^, NADH, acetyl-CoA and pyruvate

The intracellular concentrations of NAD^+^ and NADH were determined with NAD^+^/NADH detection kit (Shanghai Cablebridge Biotechnology Co. Ltd) according to the manufacturer's instructions. The intracellular acetyl-CoA was measured by Acetyl-CoA kit (Suzhou Comin Biotechnology Co. Ltd) according to the manufacturer's instructions. The intracellular concentrations of pyruvate were measured by Pyruvate Assay Kit (Suzhou Comin Biotechnology Co. Ltd) according to the manufacturer's protocols. For metabolites measurements, cell metabolites were extracted with lysate buffer provided in each kit.

### Xenograft mice studies

The BALB/c Nude mice were purchased from Beijing Vital River Laboratory Animal Technology Co., Ltd. (Beijing, China). The mice were handled in accordance with the guidance for the local care and use of laboratory animals under specific pathogen free conditions. All procedures were approved by the Animal Care and Use Committee of Wuhan Hospital of Traditional Chinese and Western Medicine (Wuhan No. 1 Hospital). The mice were then injected subcutaneously with 1 × 10^6^ HeLa cells and 0.1 ml matrix gel. When the mice grew about 10 days until the tumor diameter reached 4–5 mm, the mice were divided into two groups (five mice/group). One group was fed with 200 mg/kg sodium pyruvate for three weeks while the other group was given the same concentration of NaCl-containing PBS every two days. This dosage of sodium pyruvate was determined in preliminary experiments that manifest tumoricidal action without any apparent cytotoxicity on normal mice. The mice were humanely sacrificed and the heart, liver, kidney, brain and tumor were immediately dissected. The weight of the resulting tumors was measured according to the approved guidelines. The SIRT1 shRNA xenograft studies were performed similar to the above experiments except 1 × 10^6^ control shRNA and SIRT1 shRNA HeLa cells were used and mice were divided into four groups (nine mice/group).

### Immunohistochemistry

The immunohistochemistry was performed as described previously (27). Tumor mass was removed from the sacrificed mice, fixed in 4% paraformaldehyde, and dehydrated stepwise with varying concentrations of ethanol. The piece of tissue material is embedded and sectioned in molten paraffin liquids. Tissue slides were de-paraffinized and heated to complete the antigen retrieval. The slides were then incubated with primary antibodies followed by the maxvision secondary antibody (Bioswamp). Detections were performed using the detection refine DAB kit (Biosharp). Nuclei were stained using hematoxylin (Beyond) before mounting.

The cervical and lung tissue samples were acquired from Wuhan No. 1 Hospital and Hubei Cancer Hospital (Tongji Medical College, Huazhong University of Science and Technology). The informed consent was collected from the patients. The procedures related to human subjects were approved by Ethic Committee of Wuhan No. 1 Hospital and Hubei Cancer Hospital.

### Quantification and statistical analysis

For quantification of western blots data, ImageJ software was used to measure the relative intensity of each band, and the relative protein levels were normalized to the relative actin levels. The MNase digestion data was quantified by Gel-Pro Analyzer 4.0. Unless otherwise indicated, data are presented as the mean ± SE from at least three biological replicates, and the differences between any two groups or multiple groups were compared by unpaired *t*-tests. *P < 0.05, **P < 0.01, ***P < 0.001, and *n.s*. indicates ‘no significance’.

## RESULTS

### Exogenous pyruvate treatment inhibits cell proliferation

**Figure 1.**
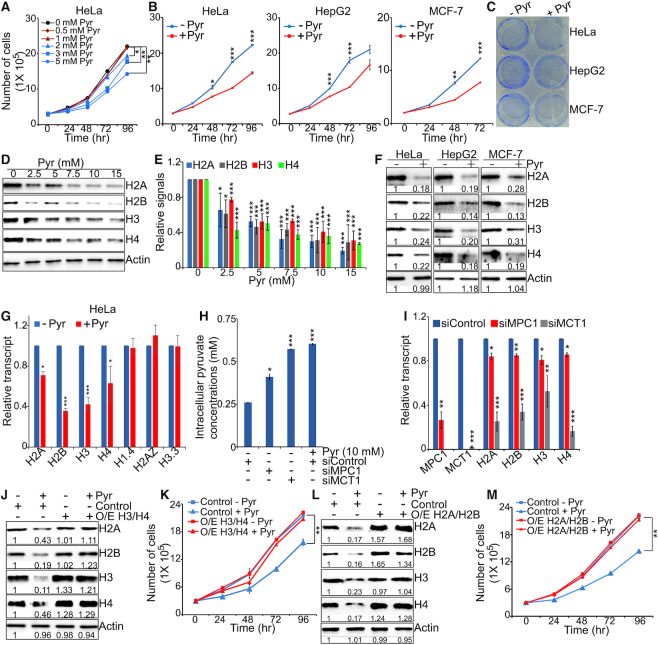
Pyruvate represses histone gene expression and inhibits cell proliferation. (**A**) Effect of pyruvate on growth of HeLa cells. Cells were treated with 0, 0.5, 1, 2, 3 and 5 mM sodium pyruvate (Pyr) and cell numbers were counted at different time points. Pyr, pyruvate. *P < 0.05; **P < 0.01. (**B**) Effect of pyruvate on growth of HeLa, HepG2 and MCF-7 cells. Cells were treated with 5 mM sodium pyruvate (+ Pyr) or 5 mM NaCl (- Pyr) and cell numbers were counted at different time points. *P < 0.05; **P < 0.01; ***P < 0.001. (**C**) Effect of pyruvate on growth of HeLa, HepG2 and MCF-7 cells. Cells were treated with 5 mM sodium pyruvate (+Pyr) or 5 mM NaCl (–Pyr) and cell growth was monitored by colony formation assays. (**D**) Effect of pyruvate on intracellular histone protein levels in HeLa cells. Cells were treated with 0–15 mM sodium pyruvate and extracted histones were analyzed by western blots with indicated antibodies. (**E**) The relative intensities of Histones/Actin in D were quantified using ImageJ with standard error (SE). Data represent the mean±SE of three independent experiments. *P < 0.05; **P < 0.01; ***P < 0.001. (**F**) Effects of pyruvate on intracellular histone protein levels in HeLa, HepG2 and MCF-7 cells. Cells were treated with 5 mM NaCl (–Pyr) or 5 mM sodium pyruvate (+Pyr) and analyzed by western blots with indicated antibodies. The relative intensity of each band was indicated. (**G**) Effect of pyruvate on histone gene transcription. HeLa cells were treated with 5 mM NaCl (–Pyr) or 5 mM sodium pyruvate (+Pyr). The transcription of histone H2A, H2B, H3 and H4 were analyzed by qRT-PCR. H1.4, H2AZ and H3.3 were used as controls. Data represent the mean ± SE of three independent experiments. *P < 0.05; ***P < 0.001. (**H**) Analysis of the intracellular pyruvate concentrations in HeLa cells transfected with siControl, siMCT1 and siMPC1. Data represent the mean ± SE (*n* = 3). siControl cells treated with 10 mM sodium pyruvate were used as controls. *P < 0.05; ***P < 0.001. (**I**) qRT-PCR analysis of histone gene transcription in HeLa cells transfected with siControl, siMCT1 and siMPC1. Data represent the mean ± SE (*n* = 3). *P < 0.05; **P < 0.01; ***P < 0.001. (**J** and **K**) Effect of pyruvate on growth of HeLa cells that overexpress histones H3 and H4. Cells were transfected with pCMV-H3/H4 (O/E H3/H4) or pCMV (Control), treated with 5 mM sodium pyruvate (+Pyr) or 5 mM NaCl (–Pyr), cell numbers were counted at different time points and histones were analyzed by western blots. **P < 0.01. (**L** and **M**) Effect of pyruvate on growth of HeLa cells that overexpress histone H2A and H2B. **P < 0.01.

To explore the impact of pyruvate on cell proliferation, we treated cells with different concentrations of sodium pyruvate for 24 h and cell proliferation was determined. Pyruvate treatment significantly reduced the proliferation of tested cells, including human cervical cancer cells (HeLa, SiHa), human liver carcinoma cells (HepG2, HCC), and human breast adenocarcinoma cells (MCF-7, MDA-MB-231) (Supplementary Figure S1A). For HeLa cells, 2 mM pyruvate can significantly inhibit cell growth (Figure 1A). To examine whether this inhibitory effect was caused by Na^+^, we treated cells with the same concentration of sodium pyruvate (+Pyr) or NaCl (–Pyr). Sodium pyruvate significantly impaired cell growth especially after 48 h when compared with NaCl treatment (Figure 1B and C). Sodium pyruvate treatment did not change the pH of the cell culture media (Supplementary Figure S1B), indicating that exogenous pyruvate inhibits cell proliferation without changing the media pH. In the following studies, sodium pyruvate instead of pyruvic acid was used for treatment.

### Pyruvate represses histone gene expression

Pyruvate is a metabolite derived from glucose and pyruvate kinase catalyzes the conversion of phosphoenolpyruvate (PEP) to pyruvate. Pyruvate kinase isoform PKM2 has been reported to play important roles in tumorigenesis by phosphorylating histone H3T11 (14). To determine whether pyruvate inhibits cell proliferation by reducing PKM2-catalyzed histone H3T11 phosphorylation (H3pT11), we treated HeLa cells with different concentrations of sodium pyruvate but found that the global H3pT11 as well as H3pT11 enrichment at histone gene promoters were not significantly affected by pyruvate treatment (Supplementary Figure S1C and D). Intriguingly, we observed that pyruvate reduced the amount of all four core histones in a dose-dependent manner (Figure 1D and E). Pyruvate also reduced histone protein levels in HepG2 and MCF-7 cells (Figure 1F). This effect was not observed when cells were treated with other glycolytic metabolites, i.e. fructose 1,6 biphosphate (FBP) and PEP (Supplementary Figure S1E and F).

To understand how exogenous pyruvate reduces histone proteins, we first examined whether pyruvate promotes histone degradation by the proteasome, which has been reported to occur during DNA repair and spermatogenesis (28). Pyruvate reduced histone proteins even in the presence of the proteasome inhibitor, MG132 (Supplementary Figure S2A), indicating that pyruvate does not affect the stability of histone proteins. Pyruvate treatment had no remarkable effect on cell apoptosis (Supplementary Figure S2B), suggesting that pyruvate reduces histone proteins not by causing cell death. Next, we investigated the effect of sodium pyruvate on histone gene transcription by quantitative reverse-transcription PCR (qRT-PCR). Similar to our western blots results (Figure 1D), pyruvate treatment significantly reduced the transcription of core histone genes but not linker histone H1.4 nor histone variants H3.3 and H2AZ (Figure 1G), suggesting that pyruvate primarily represses core histone gene expression at the transcription level.

We also examined the effect of endogenous pyruvate on histone gene expression. Pyruvate has been reported to be transported out of the cell by monocarboxylate transporter 1 (MCT1) and inhibition of MCT1 impairs tumor growth (29). We thus examined the effect of MCT1 knockdown via RNA interference (RNAi) on intracellular pyruvate levels and histone gene expression. Consistent with the reported results (29), the intracellular pyruvate concentration was significantly increased in MCT1 siRNA-treated cells, which is similar to 10 mM sodium pyruvate treatment in scrambled siRNA-treated cells (Figure 1H). Histone gene transcription was significantly reduced in MCT1 siRNA-treated cells (Figure 1I). Moreover, we also knocked down the expression of MPC1, which encodes the mitochondrial pyruvate carrier that transports pyruvate into the mitochondria for oxidative phosphorylation. In MPC1 siRNA-treated cells, the intracellular pyruvate was also significantly increased and histone genes were significantly reduced (Figure 1H and I). Interestingly, we found that there is an inverse correlation between intracellular pyruvate concentrations and histone gene expression: the intracellular pyruvate level in MCT1 knockdown cells was significantly higher than that in MPC1 knockdown cells (Figure 1H), while the transcription of histones in MCT1 knockdown cells was much lower than that in MPC1 knockdown cells (Figure 1I), similar to our western blots data that pyruvate reduces histone proteins in a dose-dependent manner (Figure 1E). Thus, both endogenous and exogenous pyruvate can repress histone gene expression.

### Overexpression of histones rescues the inhibitory effect of pyruvate on cell growth

To determine whether pyruvate inhibits cell proliferation by repressing histone gene expression, we individually transfected HeLa cells with pCMV-H3 and pCMV-H4 vectors that express histones H3 and H4 under the CMV promoter, respectively. Ectopic expression of histone H3 or H4 partially rescued pyruvate-reduced cell viability (Supplementary Figure S2C and D). We also transfected HeLa cells with pCMV-H3/H4 vectors to simultaneously express histones H3 and H4. Overexpression of H3 and H4 (O/E H3/H4) rescued pyruvate-reduced cell growth (Figure 1J and K). The slight increase of H2A and H2B could be due to coordinated expression of histones. Similar rescue effect was observed when histones H2A and H2B were overexpressed (Figure 1L and M). Together, these data suggest that pyruvate inhibits cell growth in part by repressing histone gene expression.

### Pyruvate but not its metabolic pathways represses histone gene expression

**Figure 2.**
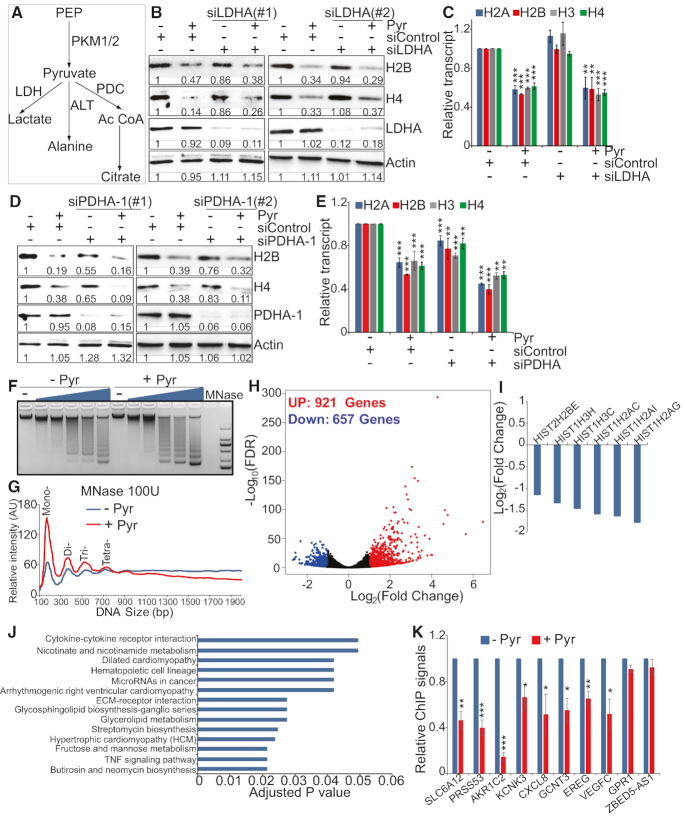
Pyruvate but not its metabolic pathways regulate histone gene expression, chromatin structure and genome-wide gene expression. (**A**) Scheme of pyruvate metabolism in cancer cells. PEP, phosphoenolpyruvate; LDH, lactate dehydrogenase; ALT, alanine transaminase; PDC, pyruvate dehydrogenase complex. (**B**) Effect of pyruvate on histone protein levels in control and LDHA knockdown cells. HeLa cells transfected with scrambled siRNA and LDHA siRNA (siLDHA#1 and siLDHA#2) were treated with 5 mM NaCl (–Pyr) or 5 mM sodium pyruvate (+Pyr). Intracellular histone proteins were analyzed by western blots. (**C**) Effect of pyruvate on histone gene transcription in control and LDHA knockdown cells as determined by qRT-PCR. Data represent the mean ± SE (*n* = 3). **P < 0.01; ***P < 0.001. (**D**) Effect of pyruvate on histone protein levels in scrambled siRNA and PDHA-1 siRNA (siPDHA-1#1 and siPDHA-1#2) transfected HeLa cells. (**E**) Effect of pyruvate on histone gene transcription in control and PDHA-1 knockdown cells as determined by qRT-PCR. Data represent the mean ± SE (*n* = 3). **P < 0.01; ***P < 0.001. (**F**) Effect of pyruvate on global chromatin structure. HeLa cells treated with or without sodium pyruvate were digested with increasing concentrations of MNase. Genomic DNA was extracted and analyzed with agarose gel electrophoresis. (**G**) Quantitation the intensity of bands for samples digested with 100 U MNase in F. (**H**) Volcano plots for differentially expressed genes by pyruvate from RNA-seq experiments. Differential expression levels of aligned sequences were calculated using significant thresholds set at fold change over two and adjusted *P* value ≤ 0.05. Red color designates significantly up-regulated genes and blue color for significantly down-regulated genes. (**I**) Histone genes were significantly down-regulated by pyruvate as determined by RNA-seq. (**J**) KEGG analysis of pathways regulated by pyruvate treatment. (**K**) Pyruvate reduced histone occupancy in 8 genes that are up-regulated by pyruvate but had little effect on two down-regulated genes as determined by ChIP analysis. *P < 0.05; **P < 0.01; ***P < 0.001.

As a glycolytic intermediate metabolite, pyruvate is formed from phosphoenolpyruvate (PEP) by pyruvate kinase (PKM1/2) and then converted to lactate and acetyl-CoA by the lactate dehydrogenase (LDH) complex and the pyruvate dehydrogenase complex (PDC), respectively (Figure 2A) (30). Pyruvate treatment had no significant effect on the expression of LDH subunit, LDHA and PDC critical subunit, PDHA-1 (Supplementary Figure S3A). To determine whether pyruvate metabolic pathways are required to repress histone gene expression, we individually knocked down the expression of LDHA and PDHA-1 in HeLa cells by siRNA and then examined the effect of pyruvate on histone gene expression. RNAi-mediated silencing of LDHA, however, did not restore pyruvate-reduced histone gene expression (Figure 2B and C). We also treated cells with lactate and no significant effect was observed on histone gene expression (Supplementary Figure S3B), indicating that LDH and LDH-catalyzed conversion of pyruvate to lactate are not required for pyruvate to repress histone gene expression.

Although knockdown of PDHA-1 by siRNA against PDHA-1 reduced histone gene expression, pyruvate still reduced histone gene expression in PDHA-1 knockdown cells (Figure 2D and E). We noticed that pyruvate reduced more histone proteins in PDHA-1 siRNA-treated cells than scrambled siRNA-treated cells (Figure 2D and E, lane 4 versus lane 2). The intracellular pyruvate concentration was significantly increased and acetyl-CoA was significantly reduced in in PDHA-1 knockdown cells compared to scrambled siRNA-treated cells (Supplementary Figure S3C), implying that the increased pyruvate accumulation or reduced acetyl-CoA in PDHA-1 knockdown cells could be responsible for the further reduced histone proteins.

Pyruvate could also be converted to alanine by alanine transaminase (ALT) (Figure 2A). However, alanine treatment has no remarkable effect on histone gene expression (Supplementary Figure S3D). Together, these data suggest that pyruvate could function as a signal molecule to repress histone gene expression.

### Pyruvate treatment alters global chromatin structure and misregulates genome-wide gene expression

As pyruvate significantly reduced global histone proteins, we next analyzed the impact of pyruvate on chromatin structure and accessibility. Micrococcal nuclease (MNase) digestion assay was used to monitor global chromatin structure changes in response to pyruvate treatment. Chromatin from pyruvate treated cells was more easily to be digested by MNase as demonstrated by the generation of more mono-, and di-nucleosomes for various concentrations of MNase (Figure 2F and G), indicative of less compact chromatin structure. The global chromatin pattern following MNase digestion is consistent with reduced histone proteins and nucleosome occupancy following pyruvate treatment.

Next, we performed RNA sequencing (RNA-seq) to assess the effect of exogenous pyruvate on the transcriptome associated with chromatin accessibility changes. More genes were significantly up-regulated (921 genes, ≥2-fold) than down-regulated (657 genes, ≤0.5-fold) by pyruvate treatment (Figure 2H), an observation that is consistent with increased chromatin accessibility (Figure 2F and G). Among these differentially expressed genes, 6 histone genes were significantly down-regulated by pyruvate treatment including *HIST2H2BE*, *HIST1H3H*, *HIST1H3C*, *HIST1H2AC*, *HIST1H2AI* and *HIST1H2AG* (Figure 2I), which is in accord with our qRT-PCR data that pyruvate significantly reduced histone gene expression (Figure 1G). Kyoto Encyclopedia of Genes and Genomes (KEGG) pathway analysis revealed that differentially regulated genes were involved in 14 different terms, including cytokine-cytokine receptor interaction, nicotinate and nicotinamide metabolism, dilated cardiomyopathy, hematopoietic cell lineage, MicroRNAs in cancer, arrhythmogenicity right ventricular cardiomyopathy, ECM-receptor interaction, glycosphingolipid biosynthesis-ganglio series, glycerolipid metabolism, etc (Figure 2J).

We randomly selected 8 genes that were up-regulated by pyruvate treatment and two genes that were down-regulated (Supplementary Figure S3E) and analyzed the effect of exogenous pyruvate on histone occupancy at these genes using chromatin immunoprecipitation (ChIP). Our data showed that pyruvate treatment significantly reduced histone H3 occupancy at eight up-regulated genes but had little effect on two down-regulated genes (Figure 2K), suggesting that pyruvate could indirectly regulate gene expression by altering nucleosome occupancy.

### Exogenous pyruvate represses histone gene expression by inducing the expression of NAMPT to increase NAD^+^ and NAD^+^/NADH

**Figure 3.**
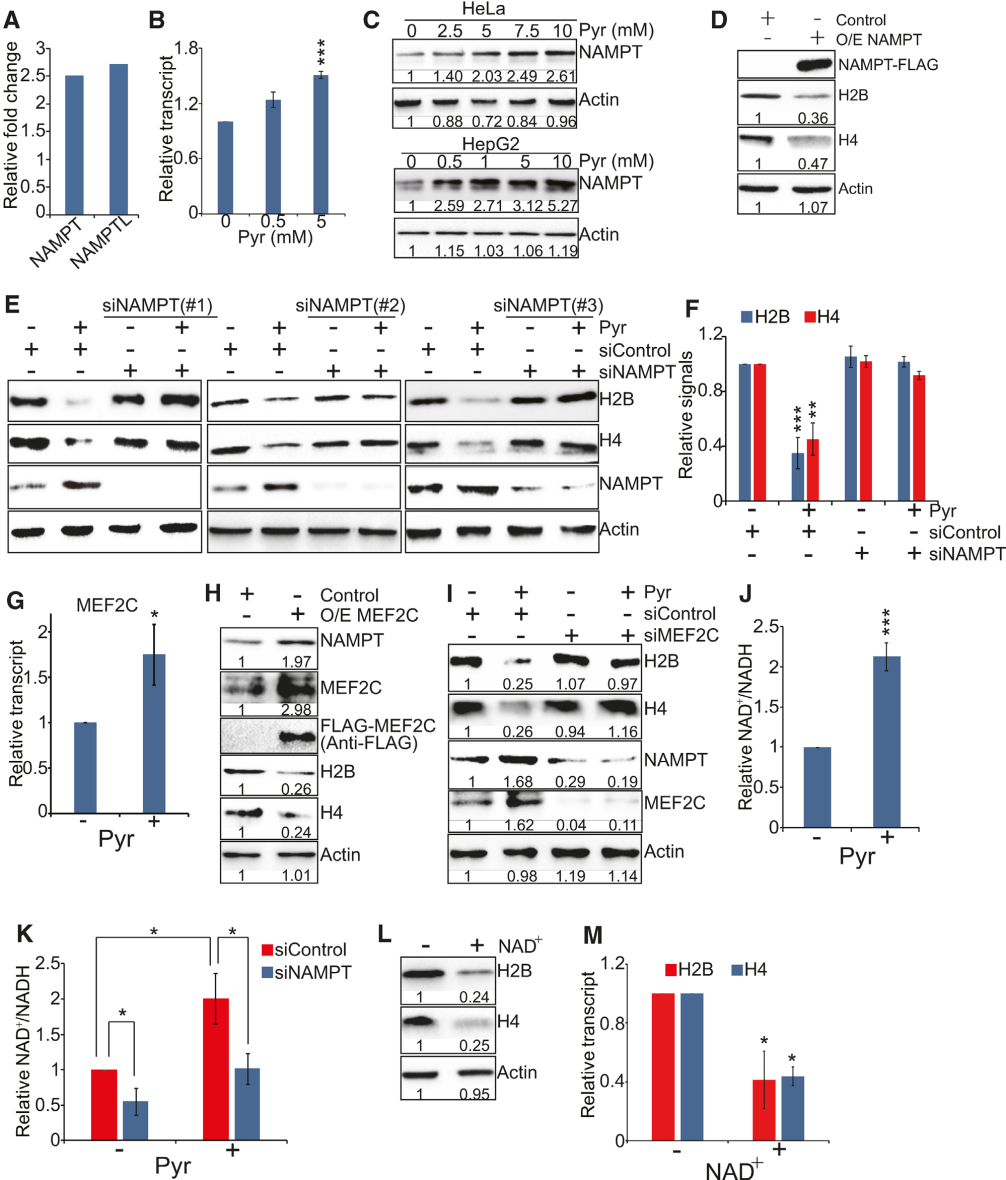
Pyruvate represses histone gene expression by inducing the expression of NAMPT and increasing the ratio of NAD^+^/NADH. (**A**) Effect of pyruvate on NAMPT and NAMPTL transcription. Data were extracted from RNA-seq analysis. (**B**) Effect of pyruvate on NAMPT transcription. HeLa cells were treated with 0, 0.5 and 5 mM sodium pyruvate (Pyr) and NAMPT transcription was analyzed by qRT-PCR. Data represent the mean ± SE (*n* = 3). ***P < 0.001. (**C**) Effect of pyruvate on NAMPT protein levels. HeLa and HepG2 cells were treated with 0–10 mM sodium pyruvate and NAMPT protein levels were analyzed by western blots. (**D**) Overexpression of NAMPT reduced intracellular histone proteins. HeLa cells were transfected with pCMV-control (Control) or pCMV-NAMPT (O/E NAMPT). Intracellular histone proteins were analyzed by western blots. (**E**) Effect of pyruvate on histone protein levels in control and NAMPT knockdown cells. HeLa cells transfected with scrambled siRNA and NAMPT siRNA (siNAMPT#1, siNAMPT#2, siNAMPT#3) were treated with 5 mM NaCl (–Pyr) or 5 mM sodium pyruvate (+Pyr). Intracellular histone proteins were analyzed by western blots. (**F**) The relative intensities of Histones/Actin in E with siNAMPT#1 were quantified using ImageJ with standard error (SE). Data represent the mean ± SE (*n* = 3). **P < 0.01; ***P < 0.001. (**G**) Effect of pyruvate on MEF2C transcription by qRT-PCR. Data represent the mean ± SE (*n* = 3). *P < 0.05. (**H**) Overexpression of MEF2C increased NAMPT expression but reduced histone gene expression. (**I**) Effect of pyruvate on histone protein levels in control and MEF2C knockdown cells. (**J**) Pyruvate significantly increased the ratio of NAD^+^/NADH. Results are means ± SE (*n* = 3). ***P < 0.001. (**K**) Effect of pyruvate on the ratio of NAD^+^/NADH in scrambled siRNA- and NAMPT siRNA-treated HeLa cells. Results are means ± SE (*n* = 3). *P < 0.05. (**L** and **M**) Effect of NAD^+^ on histone gene expression as determined by western blots (L) and qRT-PCR (M). HeLa cells were treated with 2 mM NAD^+^. Results are means ± SE (*n* = 3). *P < 0.05.

Our RNA-seq data showed that exogenous pyruvate significantly up-regulates the transcription of genes involved in nicotinate and nicotinamide metabolism, including nicotinamide phosphoribosyltransferase *NAMPT* and *NAMPTL* (Figure 3A). In mammals, 80% of nicotinamide adenine dinucleotide (NAD^+^) is derived from a two-step biosynthesis: NAMPT converts nicotinamide to nicotinamide mononucleotide (NMN), which is then converted to NAD^+^ by nicotinamide mononucleotide adenylyl-transferases (NMNAT) (31). NAMPT is a critical factor in NAD^+^ biosynthesis and knockdown of NAMPT significantly reduced the ratio of NAD^+^/NADH (Supplementary Figure S4A). First, we confirmed that exogenous pyruvate significantly induced the expression of NAMPT in HeLa cells by qRT-PCR (Figure 3B) and western blots (Figure 3C, Supplementary Figure S4B). Overexpression of NAMPT under the CMV promoter reduced intracellular histone proteins (Figure 3D), suggesting that up-regulated NAMPT inhibits histone gene expression. To investigate whether exogenous pyruvate repressed histone gene expression by inducing NAMPT expression, we examined the effect of pyruvate treatment on histone gene expression in scrambled siRNA versus NAMPT siRNA treated cells. Pyruvate significantly reduced histone proteins in scrambled siRNA treated cells but not in NAMPT knockdown cells (Figure 3E and F). These data indicate that pyruvate up-regulates NAMPT, which then represses histone gene expression.

Myocyte enhancer factor 2C (MEF2C) has been reported to function as a transcription factor for NAMPT (32). NAMPT promoter contains two MEF2C binding sites (32). We therefore examined whether pyruvate induces NAMPT transcription via MEF2C. First, we found that pyruvate significantly up-regulates the transcription of MEF2C (Figure 3G). Overexpression of MEF2C increased NAMPT expression and reduced histone gene expression (Figure 3H). Knockdown of MEF2C reduced NAMPT expression (Figure 3I). Moreover, pyruvate did not induce the expression of NAMPT in MEF2C siRNA-transfected cells and MEF2C knockdown rescued the inhibitory effect of pyruvate on histone gene expression (Figure 3I), similar to the results in NAMPT knockdown cells (Figure 3E and F). Together, these data indicate that pyruvate induces NAMPT expression in a MEF2C-dependent manner.

As NAMPT is required for NAD^+^ biosynthesis, we next investigated the effect of exogenous pyruvate on NAD^+^ and the ratio of NAD^+^/NADH. Pyruvate treatment significantly increased both NAD^+^ and NAD^+^/NADH ratio (Figure 3J, Supplementary Figure S4C); however, pyruvate-induced NAD^+^ and NAD^+^/NADH were significantly reduced in NAMPT knockdown cells when compared with control cells (Figure 3K, Supplementary Figure S4C), indicating that exogenous pyruvate up-regulates NAMPT to increase the ratio of NAD^+^/NADH. To determine whether pyruvate reduced histone gene expression by increasing NAD^+^ biosynthesis and NAD^+^/NADH, we treated cells with NAD^+^ and then analyzed its impact on histone gene expression by western blots. Similar to pyruvate treatment (Figure 1D and E), exogenous NAD^+^ treatment significantly repressed histone gene expression (Figure 3L and M). All these data indicate that exogenous pyruvate represses histone gene expression primarily by inducing the expression of NAMPT, which then promotes NAD^+^ biosynthesis and increases the ratio of NAD^+^/NADH.

### Exogenous pyruvate activates SIRT1 to deacetylate histones and repress histone gene expression

**Figure 4.**
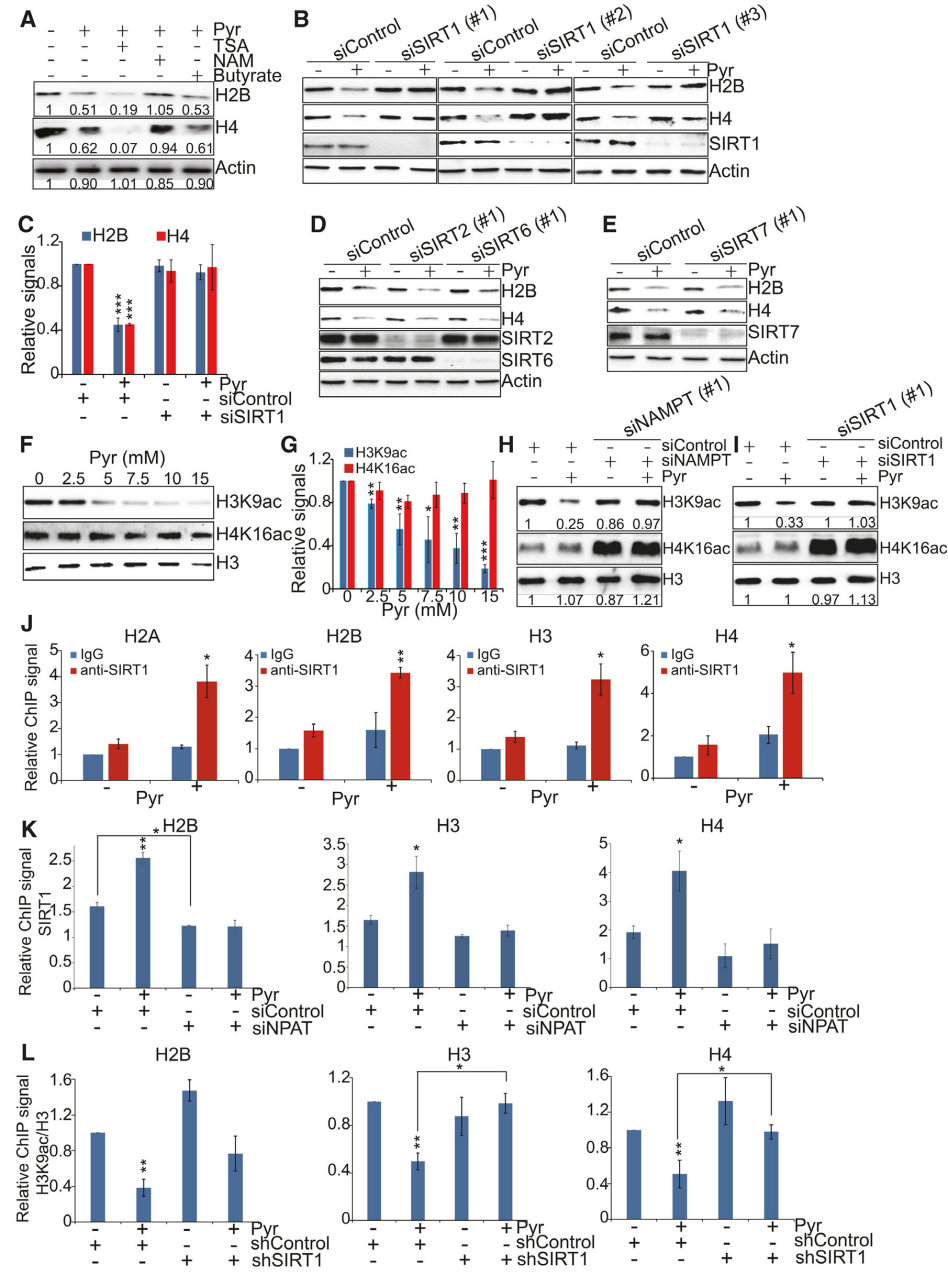
Pyruvate represses histone gene expression by enhancing SIRT1 activity and binding at histone gene promoters. (**A**) Western blots analysis of histone proteins in HeLa cells treated with sodium pyruvate (Pyr) together with TSA, nicotinamide, or sodium butyrate. (**B**) Effect of pyruvate on histone proteins in control and SIRT1 knockdown cells. HeLa cells transfected with scrambled siRNA (siControl) and SIRT1 siRNA (siSIRT1#1, siSIRT1#2, siSIRT1#3) were treated with 5 mM NaCl (–Pyr) or 5 mM sodium pyruvate (+Pyr). Intracellular histone proteins were analyzed by western blots. (**C**) The relative intensities of Histones/Actin in B with siSIRT1#1 were quantified using ImageJ with standard error (SE). Data represent the mean ± SE of three independent experiments. ***P < 0.001. (**D** and **E**) Effect of pyruvate on histone proteins in scrambled siRNA, SIRT2 siRNA, SIRT6 siRNA and SIRT7 siRNA transfected HeLa cells as determined by western blots. (**F**) Effect of pyruvate on H3K9ac and H4K16ac in HeLa cells by western blots. HeLa cells were treated with 0–15 mM sodium pyruvate. Histones were extracted and H3K9ac and H4K16ac were examined by western blots with indicated antibodies. (**G**) The relative intensities of H3K9ac/H3 and H4K16ac/H3 in Figure 4F were quantified using ImageJ with standard error (SE). Data represent the mean±SE of three independent experiments. *P < 0.05; **P < 0.01; ***P < 0.001. (**H**) Effect of pyruvate on H3K9ac and H4K16ac in control and NAMPT knockdown cells. HeLa cells transfected with scrambled siRNA (siControl) and NAMPT siRNA (siNAMPT) were treated with 5 mM NaCl (–Pyr) or 5 mM sodium pyruvate (+Pyr). Histones were extracted and histone modifications were analyzed by western blots. (I) Effect of pyruvate on H3K9ac and H4K16ac in control and SIRT1 knockdown cells as determined by western blots. (**J**) Pyruvate enhanced SIRT1 binding at histone H2A, H2B, H3 and H4 promoter regions by ChIP in HeLa cells. IgG was used as negative controls. Results are means ± SE (*n* = 3). *P < 0.05; **P < 0.01. (**K**) ChIP analysis of the effect of pyruvate on SIRT1 binding at histone H2B, H3 and H4 promoters in Control and NPAT knockdown HeLa cells. Results are means ± SE (*n* = 3). *P < 0.05; **P < 0.01. (**L**) ChIP analysis of the effect of pyruvate on the ratio of H3K9ac/H3 at histone H2B, H3 and H4 promoters in shControl and shSIRT1 HeLa cells. Results are means ± SE (*n* = 3). *P < 0.05; **P < 0.01.

How does exogenous pyruvate repress histone gene expression by promoting NAD^+^ biosynthesis and increasing NAD^+^/NADH ratio? Sirtuins (SIRT1-7) are NAD^+^-dependent protein deacetylases and some sirtuins, i.e. SIRT1, -mediated histone deacetylation is generally associated with transcription repression (33). Since the activity of sirtuins relies on NAD^+^ (34), we examined whether sirtuins are required for pyruvate to repress histone gene expression. HeLa cells were treated with sodium pyruvate along with the sirtuins inhibitor, nicotinamide (NAM). As controls, other histone deacetylase inhibitors were also examined, including trichostatin A (TSA) and sodium butyrate. Our data showed that NAM but not TSA or sodium butyrate restored histone gene expression levels that were reduced by pyruvate (Figure 4A, Supplementary Figure S5A). NAM restored histone gene expression in pyruvate-treated cells in a wide range of concentrations (Supplementary Figure S5B), suggesting that sirtuin(s) is required for pyruvate to repress histone gene expression.

There are 7 sirtuins in mammals, SIRT1-7: SIRT1, SIRT6 and SIRT7 localize in the nucleus; SIRT2 shuttles between the nucleus and the cytoplasm; SIRT3, SIRT4 and SIRT5 localize in the mitochondria (35). To investigate which sirtuin(s) plays a critical role in pyruvate-repressed histone gene expression, we individually knocked down the expression of four nuclear-localized sirtuins, SIRT1, SIRT2, SIRT6 and SIRT7 in HeLa cells and then analyzed the effect of exogenous pyruvate on intracellular histone protein levels. Although pyruvate treatment reduced histone proteins in SIRT2, SIRT6 and SIRT7 knockdown cells, histones were not reduced by exogenous pyruvate in SIRT1 knockdown cells (Figure 4B–E, Supplementary Figure S5C and D). These data indicate that SIRT1 is specifically required for exogenous pyruvate to repress histone gene expression. Pyruvate has been shown to inhibit the activity of HDAC1 and HDAC3 (36); however, pyruvate still reduced histone proteins in HDAC1 and HDAC3 knockdown cells (Supplementary Figure S5E), indicating that HDAC1 and HDAC3 are not involved in pyruvate-repressed histone gene expression.

One important deacetylation target of SIRT1 is histone H3K9 acetylation (H3K9ac), which is required for gene expression (37,38). Pyruvate significantly reduced H3K9ac in a dose-dependent manner in HeLa cells (Figure 4F and G), consistent with our data that pyruvate activates SIRT1. Although SIRT1 can also deacetylate H4K16 (39), pyruvate did not significantly reduce H4K16ac (Figure 4F and G). Pyruvate did not change the global levels of SIRT1 (Supplementary Figure S5F), indicating that exogenous pyruvate primarily enhances the activity of SIRT1 but not its expression. Resveratrol has been reported to increase NAD^+^ and the ratio of NAD^+^/NADH and activate the histone deacetylase activity of SIRT1 (40). We thus treated cells with resveratrol and found it reduced H3K9ac as well as histone protein levels (Supplementary Figure S5G), consistent with our finding that activated SIRT1 represses histone gene expression.

To determine whether exogenous pyruvate activates SIRT1 by inducing the expression of NAMPT, we then examined the impact of pyruvate treatment on H3K9ac in NAMPT or SIRT1 knockdown cells. Our data showed that pyruvate-reduced H3K9ac was partly rescued in NAMPT knockdown cells (Figure 4H, Supplementary Figure S5H) and SIRT1 knockdown cells (Figure 4I, Supplementary Figure S5I). In budding yeast, there was a significant reduction of histones in H3K9A mutant but not in H4K16R mutant when compared with its wild-type counterpart (Supplementary Figure S5J), indicating that H3K9ac promotes histone gene expression, which is consistent with the reported role of histone acetyltransferase Gcn5 in histone gene transcription (41).

To show that SIRT1 directly represses histone gene expression, we examined the binding of SIRT1 at histone genes by ChIP analysis. Our data showed that SIRT1 preferentially binds to histone gene promoters rather than their coding regions (Supplementary Figure S6A). We also examined the effect of exogenous pyruvate on SIRT1 binding at histone gene promoters and found that pyruvate remarkably enhanced the binding of SIRT1 at histone gene promoters (Figure 4J). As controls, pyruvate did not stimulate the binding of SIRT1 at other non-histone genes that are repressed by pyruvate, i.e. GPR1, ZBED-AS1 and NPNT (Supplementary Figure S6B).

As a crucial factor in regulating histone gene expression, nuclear protein ataxia-telangiectasia (NPAT) has been shown to recruit transcription regulators to histone genes, including histone acetyltransferase Tip60 complex (42). As SIRT1 interacts with Tip60 (43), we thus examined the effect of NPAT on SIRT1 occupancy at histone genes. Knockdown of NPAT significantly reduced SIRT1 binding at histone gene promoters (Figure 4K). Moreover, pyruvate enhanced SIRT1 binding at histone gene promoters in scrambled siRNA treated cells but not in NPAT siRNA treated cells (Figure 4K), indicating that pyruvate enhances the binding of SIRT1 at histone genes in a NPAT-dependent manner.

Next, we examined H3K9ac at histone gene promoters upon pyruvate treatment and found that pyruvate significantly reduced H3K9ac at histone gene promoters (Supplementary Figure S6C). Moreover, we examined the effect of pyruvate on H3K9ac at histone gene promoters in SIRT1 knockdown cells by ChIP. Although pyruvate reduced H3K9ac at histone promoters in control shRNA cells, knockdown of SIRT1 rescued pyruvate-reduced H3K9ac at histone gene promoters (Figure 4L, Supplementary Figure S6D), indicating that pyruvate enhances the binding of SIRT1 at histone gene promoters to deacetylate H3K9. As a control, pyruvate did not reduce H4K16ac at histone genes (Supplementary Figure S6E). We also examined whether NAMPT binds to histone gene promoters by ChIP analysis. However, no good ChIP signals were observed for NAMPT.

Collectively, our data showed that pyruvate reduces H3K9ac by enhancing the binding and activity of SIRT1 at histone gene promoters via NAMPT and NAD^+^.

### Exogenous pyruvate represses histone gene expression independent of cell cycle

**Figure 5.**
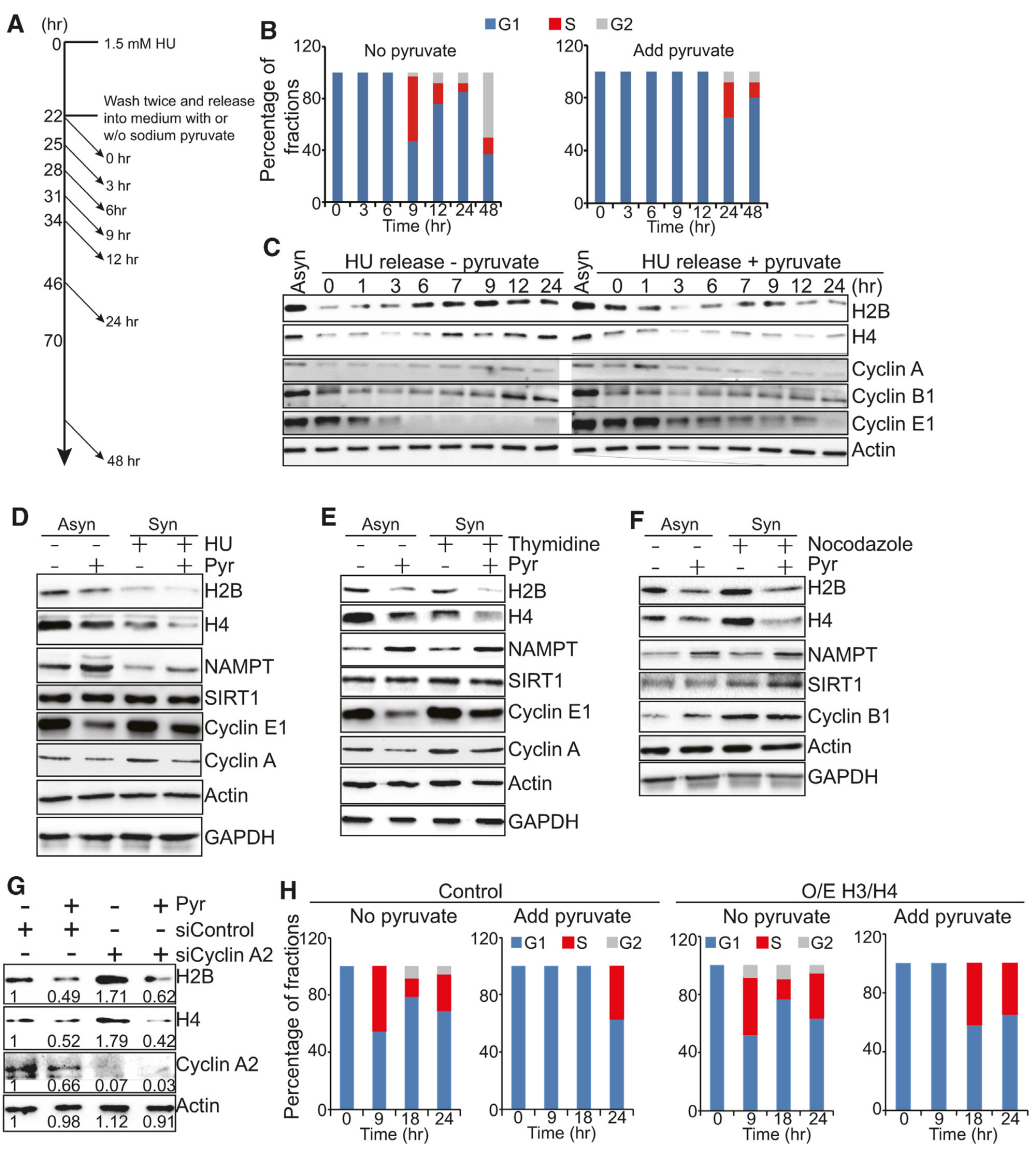
Pyruvate represses histone gene expression not by causing cell cycle arrest. (**A**–**C**) Pyruvate caused cell cycle arrest at G1/S phase. Cells were synchronized in 1.5 mM HU for 22 h. Cells were washed twice and then released into medium with or without sodium pyruvate. Cells were taken at different time points and the cell cycle progression was determined by flow cytometry (B) and western blots (C). The expression of Cyclin A begins at late G1 and reaches the maximal level during the G2 phase (54). The level of Cyclin B1 is minimal at G1, increase at S phase and peaks at the G2/M phase (55). The expression of Cyclin E1 starts in the middle of the G1 phase and ends at the middle of the S phase (54). Asyn, asynchronized cells. (**D**–**F**) Pyruvate reduced histone proteins in HU-, thymidine- and nocodazole-synchronized HeLa cells. Asynchronized HeLa cells were used as a control. Asyn, asynchronized cells; Syn, synchronized cells. (**G**) Effect of pyruvate on histone proteins in control and Cyclin A2 knockdown HeLa cells. (**H**) Overexpression of histones H3 and H4 partly rescued pyruvate-induced cell cycle arrest. HeLa cells transfected with control pCMV (Control) and pCMV-H3/H4 (O/E H3/H4) plasmids were synchronized with HU and then released into medium with or without sodium pyruvate treatment. Cells were taken at different time points to determine the cell cycle progression by flow cytometry.

Histone gene expression is tightly coupled to cell cycle progression with synthesis and accumulation of histones restricted to S phase (44). Reduced expression or depletion of core histones during DNA replication delays S phase completion and results in mitotic arrest (45). We thus examined the effect of pyruvate on cell cycle progression. Cells were synchronized to G1/S phase with hydroxyurea (HU) (46), washed twice and then released into medium with or without sodium pyruvate treatment. Cells were harvested at different time points and cell cycle profiles were analyzed by flow cytometry (Figure 5A). In the absence of pyruvate treatment, cell cycle progressed normally to S phase when histones were largely synthesized (Figure 5B and C). However, in the presence of exogenous pyruvate, cells were delayed entry into S phase and blocked in G1/S with few histones detected (Figure 5B and C).

To examine whether pyruvate reduced histone gene expression by causing cell cycle arrest, we performed a time-course experiment of pyruvate treatment, measuring the transcription of histone genes at each time point by qRT-PCR. We also examined the transcription of P21 (CDKN1A) and P57 (CDKN1C), which are important for G1/S and G2/M transition (47). Histone genes were significantly repressed by pyruvate as early as 0.5 h, whereas P21 and P57 were not induced at the time tested (Supplementary Figure S7A), suggesting that pyruvate represses histone genes prior to cell cycle arrest. To further confirm that, we individually synchronized cells at G1/S phase by HU, early S phase by double thymidine block and G2/M phase by nocodazole (48). These synchronized cells were then treated with sodium pyruvate. Pyruvate treatment significantly reduced histone gene expression in both asynchronized and HU-synchronized cells (Figure 5D, Supplementary Figure S7B), double thymidine-synchronized cells (Figure 5E, Supplementary Figure S7C), and nocodazole-synchronized cells (Figure 5F, Supplementary Figure S7D), indicating that pyruvate represses histone gene expression not by causing cell cycle arrest. Moreover, pyruvate induced NAMPT expression in HU-, thymidine-, and nocodazole-synchronized HeLa cells (Figure 5D–F, Supplementary Figure S7B–D), further confirming that pyruvate induces the expression of NAMPT to repress histone gene expression in a cell-cycle independent manner. We also knocked down the expression of cyclin A2 to cause cells to arrest in G2/M phase (49) and found that pyruvate still repressed histone gene expression in cyclin A2 knockdown cells (Figure 5G).

To examine whether pyruvate caused cell cycle arrest by repressing histone gene expression, we transfected cells with the empty vector or vectors that overexpress histone H3 and H4 driven by the CMV promoter (pCMV-H3/H4) (Supplementary Figure S7E) and then examined cell cycle profiles when treated with or without pyruvate. Overexpression of H3 and H4 partly, if not totally, rescued the delayed S phase entry by pyruvate (Figure 5H). As reduced histone expression impairs cell entry into S phase (45), these data suggest that exogenous pyruvate delays cell entry into S phase in part by repressing histone gene expression.

### Exogenous pyruvate inhibits cell proliferation and tumor growth via the NAMPT-NAD^+^-SIRT1 pathway

**Figure 6.**
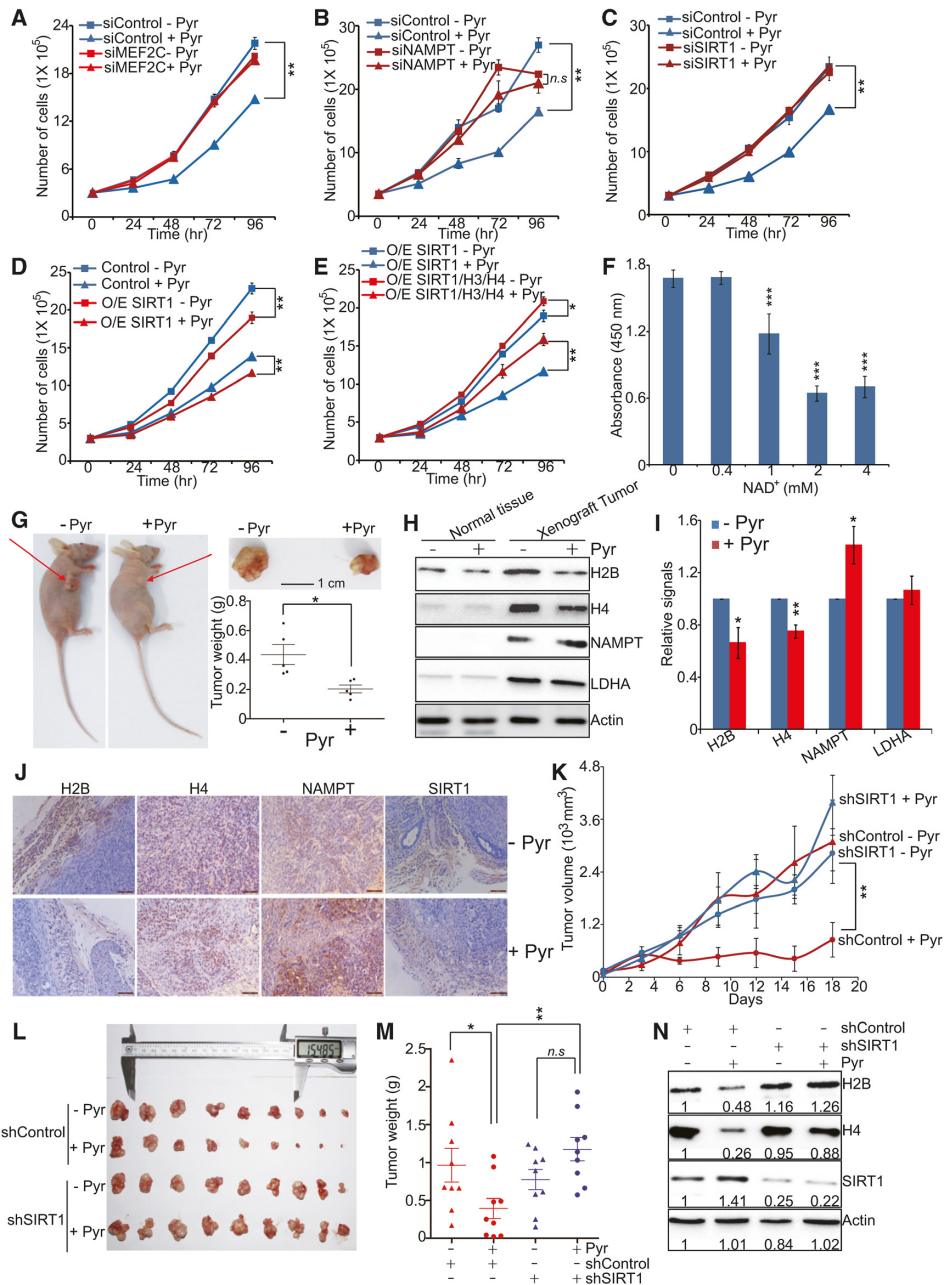
Exogenous pyruvate inhibits cell proliferation and tumor growth via the NAMPT-NAD^+^-SIRT1 pathway. (**A**) Effect of pyruvate on the growth of control and MEF2C knockdown cells. HeLa cells transfected with scrambled siRNA (siControl) and MEF2C siRNA (siMEF2C) were treated with 5 mM sodium pyruvate (+ Pyr) or 5 mM NaCl (- Pyr) and cell numbers were counted at different time points. **P< 0.01. (**B**) Effect of pyruvate on the growth of control and NAMPT knockdown cells. **P < 0.01; *n.s*, no significance. (**C**) Effect of pyruvate on the growth of control and SIRT1 knockdown cells. **P < 0.01. (**D**) Effect of pyruvate on the growth of HeLa cells that overexpress SIRT1. Cells were transfected with pCMV-SIRT1 (O/E SIRT1) or pCMV (Control), treated with 5 mM sodium pyruvate (+Pyr) or 5 mM NaCl (–Pyr) and cell numbers were counted at different time points. **P < 0.01. (**E**) Effect of pyruvate on the growth of HeLa cells that overexpress SIRT1 (O/E SIRT1) or simultaneously express SIRT1 and histones (O/E SIRT1/H3/H4). *P < 0.05; **P < 0.01. (**F**) Effect of NAD^+^ on the growth of HeLa cells. ***P < 0.001. (**G**) Pyruvate significantly reduced xenograft tumor growth. Nude mice injected with HeLa cells were administered with PBS (–Pyr) or 200 mg/kg sodium pyruvate (+Pyr) (five mice/group). The xenograft tumors were dissected at the endpoint. Shown are the representative images of mice and dissected tumors. Scale bar: 1 cm. Final weight of tumors from PBS and pyruvate-treated mice was plotted in the form of the mean tumor weight ± S.E. of five mice per group. *P < 0.05. (**H**) Western blots of histones, NAMPT, LDHA and Actin in mice normal tissues and xenograft tumors administrated with PBS or sodium pyruvate. (**I**) Quantitation of western blots data in H. Data represent means ± SE (*n* = 3). *P < 0.05; **P < 0.01. (**J**) Representative IHC staining images of histones, NAMPT and SIRT1 in xenograft tumors administered with PBS or pyruvate. (K-M) Effect of pyruvate on xenograft growth of control (control shRNA) and SIRT1 knockdown (shSIRT1) cells. Xenograft mice were administrated with PBS (–Pyr) or sodium pyruvate (+Pyr) (nine mice/group). The xenograft tumors were measured over time and dissected at the endpoint. Quantification of the average volume of tumors over time was shown in (**K**). The dissected tumors were shown in (**L**). The quantification of the tumor weight was shown in (**M**). *P < 0.05; **P < 0.01; *n.s*., no significance. (**N**) Western blots of histones, SIRT1 and actin in control shRNA and SIRT1 shRNA tumors administered with PBS (–Pyr) or sodium pyruvate (+Pyr) in L. Shown is the typic example of 3 independent experiments.

To show that whether exogenous pyruvate inhibits cell proliferation by repressing histone gene expression via the NAMPT-NAD^+^-SIRT1 pathway, we first examined the effect of pyruvate on the growth of MEF2C knockdown cells. Pyruvate significantly inhibited the growth of control cells; however, it has no significant effect on MEF2C siRNA transfected cells (Figure 6A). Similar to MEF2C knockdown cells, pyruvate showed no significantly inhibitory effect on the growth of NAMPT or SIRT1 knockdown cells (Figure 6B and C; Supplementary Figure S8A). Moreover, overexpression of SIRT1 enhanced the inhibitory effect of pyruvate on histone gene expression and cell growth (Figure 6D, Supplementary Figure S8B) and this enhanced effect was partly ameliorated by overexpression of histones (Figure 6E), suggesting that other non-histone targets of SIRT1 may work to repress cell growth when SIRT1 is overexpressed. As the SIRT1 co-activator, NAD^+^ inhibited the proliferation of HeLa cells in a dose-dependent manner (Figure 6F). Resveratrol has been shown to repress histone gene expression and a combined treatment of pyruvate and resveratrol had greater inhibitory effects on cell proliferation when compared to pyruvate treatment alone (Supplementary Figure S8C), further corroborating that reducing histone gene expression inhibits cell growth. SIRT1 has been reported to deacetylate P53 to regulate cell proliferation (50). We thus examined the effect of pyruvate on growth of P53 knockdown cells. Pyruvate repressed histone gene expression and cell proliferation in P53 knockdown cells (Supplementary Figure S8D and E), indicating that pyruvate inhibits cell growth independent of P53 pathway.

To investigate the effects of exogenous pyruvate on HeLa cell growth and tumorigenicity in vivo, HeLa cells were injected into nude mice. After tumors had developed, the mice were administered with 200 mg/kg sodium pyruvate or the same concentration of NaCl-containing PBS, respectively. Administration of this dosage of pyruvate did not affect the body weight and food intake of the mice (Supplementary Figure S8F and G). Pyruvate administration significantly increased the concentrations of pyruvate inside the kidney without influencing the body weight of xenografted mice (Supplementary Figure S8H and I). Pyruvate administration significantly reduced the tumor weight when compared with PBS controls (Figure 6G). The histone protein levels were remarkably reduced by pyruvate administration in tumors but not in normal mice tissues (Figure 6H and I). Further analysis of histone mRNA levels revealed that pyruvate administration significantly repressed histone gene transcription (Supplementary Figure S8J). We also found both mRNA and protein levels of NAMPT were significantly increased by pyruvate in tumors (Figure 6H and I, Supplementary Figure S8J). To further define the relevance of histone expression and NAMPT upon pyruvate treatment, we used immunohistochemistry (IHC) staining analysis. The levels of histones were reduced and NAMPT was increased in pyruvate-treated tumors (Figure 6J). The levels of SIRT1 were not changed by pyruvate treatment in tumors (Figure 6J), which is consistent with our in vitro data that pyruvate enhances SIRT1 activity but not its expression (Supplementary Figure S5F).

We then examined whether SIRT1 is required for pyruvate to repress tumorigenesis by injecting control shRNA and stable SIRT1 knockdown (SIRT1 shRNA) HeLa cells into nude mice. After tumors had developed, the mice were administrated with PBS or sodium pyruvate. While exogenous pyruvate significantly inhibited the growth of control shRNA cells in xenograft mice, pyruvate treatment did not reduce the tumorigenesis of SIRT1 shRNA cells (Figure 6K–M). By analyzing histone proteins in tumors developed from control shRNA and SIRT1 shRNA HeLa cells, we found that while pyruvate treatment reduced histone proteins in control shRNA-derived tumors, histone proteins were not reduced by pyruvate in SIRT1 shRNA-derived tumors (Figure 6N). Together, these data indicate that exogenous pyruvate activates SIRT1 to repress histone gene expression and inhibit tumorigenesis.

### The pyruvate concentration inversely correlates with histone protein levels in cancer patients

**Figure 7.**
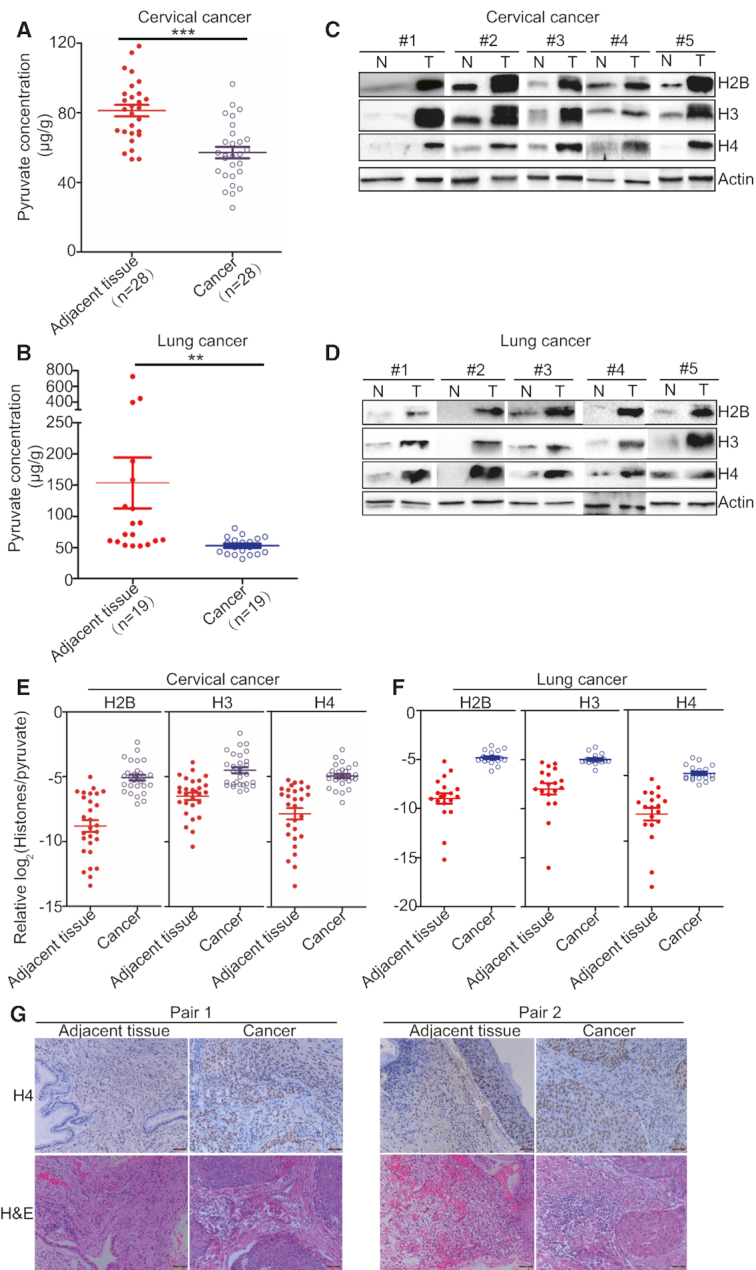
The pyruvate concentration negatively correlates with histone expression levels in cancer tissues. (**A**) The pyruvate concentration is significantly lower in cancer tissues than adjacent normal tissues from 28 cervical cancer patients. The pyruvate concentration was calculated as μg pyruvate per tissue weight (g). ***P < 0.001. (**B**) The pyruvate concentration is significantly lower in cancer tissues than adjacent normal tissues from 19 lung cancer patients. **P < 0.01. (**C**) Analysis of histone proteins in paired tissue specimens of cervical cancer (T) and adjacent normal tissues (N). A total of 28 paired tissue specimens were examined and only 5 paired tissue specimens were shown here. See Figure S9A for western blots data of all 28 samples. (**D**) Analysis of histone proteins in paired tissue specimens of lung cancer (T) and adjacent normal tissues (N). A total of 19 paired tissue specimens were examined and only 5 paired tissue specimens were shown here. See Figure S10A for western blots data of all 19 samples. (**E**) Cervical tumor tissues have higher histones/pyruvate than adjacent normal tissues. The histone protein levels (normalized to Actin) were obtained by quantitating the western blots data for 28 paired tissue specimens in Figure S9A with ImageJ. The pyruvate concentration data were derived from A. (**F**) Lung tumor tissues have higher histones/pyruvate than adjacent normal tissues. The histone protein levels (normalized to Actin) were obtained by quantitating the western blots data for 19 paired tissue specimens in Figure S10A with ImageJ. The pyruvate concentration data were derived from B. (**G**) Immunohistochemical staining of histone H4 expression in cervical cancer patients. Only 2 pairs were shown here. Scale bar: 50 μm.

To assess the clinical relevance of our finding that pyruvate represses histone gene expression to inhibit cell proliferation and tumor growth, we examined the correlation between the pyruvate concentration and histone expression levels in cancer patients. We measured the concentration of pyruvate in 28 pairs of cervical tumor tissues and adjacent normal tissues from cervical cancer patients. The pyruvate concentration in cervical cancer tissues is 57.20 ± 3.25 μg/g wet weight, which is significantly lower than that in adjacent normal tissues (81.24 ± 3.31 μg/g wet weight) (Figure 7A). We also found that the pyruvate concentration in lung cancer tissues (51.73 ± 3.14 μg/g wet weight) is significantly lower than that in adjacent normal tissues (160.92 ± 40.23 μg/g wet weight) (Figure 7B). Next, we determined histone proteins in the same pairs of tumor and adjacent tissues by western blots and immunohistochemical (IHC) staining. Western blots analysis showed that histone proteins were much higher in tumor (T) than those in adjacent non-cancerous background tissues (N) derived from cervical cancer patients (Figure 7C, Supplementary Figure S9A) and lung cancer patients (Figure 7D, Supplementary Figure S10A). Moreover, tumor tissues have significantly higher histones/pyruvate ratio than adjacent normal tissues (Figure 7E and F). There is an inverse correlation between pyruvate concentrations and histone protein levels in cancer tissues and adjacent non-cancerous tissues: cancer tissues have relatively higher histone protein levels but lower pyruvate concentration, while adjacent non-cancerous tissues have relatively lower histone protein levels but higher pyruvate concentration (Supplementary Figures S9B and 10B). IHC staining showed that cancer tissues had remarkably intense staining and adjacent non-cancerous tissues had either weak or moderate staining (Figure 7G), further supporting that histone proteins are highly expressed in cancer.

We also analyzed the transcriptome data of cervical squamous cell carcinoma and endocervical adenocarcinoma from The Cancer Genome Atlas (TCGA) and found that most histone genes were significantly up-regulated in cervical cancer tissues compared with normal tissues (Supplementary Figure S11A). Histone genes were also up-regulated in other cancers, including liver hepatocellular carcinoma (Supplementary Figure S11B), breast invasive carcinoma (Supplementary Figure S11C), and lung adenocarcinoma (Supplementary Figure S11D). Collectively, these results strongly support a negative correlation between pyruvate and histone proteins in both cervical cancer and lung cancer.

## DISCUSSION

In this study, we report that exogenous pyruvate represses histone gene expression and inhibits cell proliferation by activating the NAMPT-NAD^+^-SIRT1 pathway (Supplementary Figure S12). Exogenous pyruvate induces the expression of NAMPT via MEF2C to promote NAD^+^ synthesis and increase the ratio of NAD^+^/NADH, which then enhances the binding and activity of SIRT1 at histone gene promoters in a NPAT-dependent manner. Activated SIRT1 deacetylates histone proteins to repress histone gene expression. As a consequence, cell cycle is arrested at G1/S and cell growth is impaired by pyruvate treatment. Therefore, our study describes a novel signaling pathway that connects cell metabolism with histone gene transcription and tumorigenesis. It will be of interest to discover whether these results are generalizable to the clinic treatment of cancers.

Pyruvate has been reported to have protective effects on cancer cells by maintaining ATP homeostasis, preventing DNA damage and facilitating hypoxia adaptation (30,51). Liu *et al.* reported that pyruvate can be converted to acetate by consuming reactive oxygen species (ROS) to maintain endogenous acetyl-CoA and lipogenesis (52). Here, we find that exogenous pyruvate causes cell cycle arrest and inhibits cell proliferation. Using a xenograft mouse model, we show that administration of pyruvate reduces tumor growth in vivo. Most importantly, pyruvate has no apparent side effects on mice tested in this study, including body weight and food intake. Although pyruvate has been shown to protect cancer cells from DNA damage and oxidative stress, the beneficial effect of pyruvate occurs in specific context, i.e. electron transport chain (ETC) deficient (30), nutritional excess and elevated glucose metabolism (52).

Our data reveal a mechanism by which exogenous pyruvate inhibits tumor growth partly if not totally by repressing histone gene expression. Some histone regulators have been involved in tumorigenesis. For example, NPAT has been shown to undergo a truncated deletion in Nodular lymphocyte predominant Hodgkin lymphoma (NLPHL) and this mutation can serve as a candidate risk factor for Hodgkin lymphoma (53). Pyruvate represses histone gene expression, which results in less compact chromatin, deregulated gene expression and cell cycle arrest. Overexpression of histones can partly rescue the delayed cell cycle progression and alleviate the inhibitory role of pyruvate on cell proliferation. Knockdown of MEF2C, NAMPT or SIRT1 alleviates the inhibitory role of pyruvate on histone gene expression and cell proliferation. Overexpression of SIRT1 enhances the inhibitory effect on cell growth and overexpression of histones can partly ameliorate SIRT1-enhanced inhibitory effect of pyruvate on cell growth. Knockdown of SIRT1 target, P53 cannot rescue the inhibitory effect of pyruvate on cell growth. Moreover, tumors have a higher ratio of histone proteins to intracellular pyruvate levels (histones/pyruvate) than adjacent normal tissues, which is consistent with our findings that pyruvate treatment represses histone gene expression. Although it is difficult to exclude all potential targets of pyruvate and SIRT1, our current data indicate that pyruvate inhibits cell growth in part by repressing histone gene expression via the NAMPT-NAD^+^-SIRT1 pathway.

Histone gene expression is regulated by histone modifications. We have previously reported that histone methyltransferase Set1-catalyzed H3K4me3 promotes histone gene expression by restricting the spread of the repressive HIR/Asf1/Rtt106 complex from histone gene promoters to histone coding regions (24). Histone acetyltransferases Gcn5 and Tip60 have been shown to regulate histone gene expression in yeast and mammals, respectively (41,42). A recent study by Gruber *et al.* showed that histone acetyltransferase 1 (HAT1) facilitates GCN5 to acetylate H3K9 to promote histone H4 expression and cell cycle progression (54). Here, we show that H3K9ac promotes histone gene expression and SIRT1 deacetylates H3K9 to repress histone gene expression. As SIRT1 has many deacetylation targets, we cannot exclude the possible functions of other acetylation sites in histone gene regulation. In addition to histone modifications, histone genes are also regulated by cell metabolism. Our data showed that exogenous pyruvate promotes NAD^+^ biosynthesis and increases the ratio of NAD^+^/NADH, which then activates SIRT1 to deacetylate H3K9. ChIP analysis showed that exogenous pyruvate increases the binding of SIRT1 at histone gene promoters in a NPAT-dependent manner. Although little is known about how HAT1 and GCN5 are recruited to histone gene promoters, it is possible that pyruvate may also affect the binding of HATs such as Tip60 to histone genes. Further efforts are required to investigate this possibility.

Although cancer cells have accelerated glycolysis, the intracellular pyruvate level is maintained at low levels. Cancer cells highly express MCT1 to mediate pyruvate export (29) and our data showed that knockdown of MCT1 significantly increased intracellular pyruvate accumulation. This could be the primary way for cancer cells to keep intracellular pyruvate low. Cancer cells also highly express enzymes to metabolize pyruvate, i.e. LDH-mediated conversion of pyruvate to lactate (55). Our data showed that pyruvate represses histone gene expression independent of LDH. Pyruvate is also converted to acetyl-CoA by pyruvate dehydrogenase (PDH) complex and knockdown of PDHA-1 significantly reduced intracellular acetyl-CoA and increased pyruvate accumulation. Although histone genes were significantly reduced in PDHA-1 knockdown cells, pyruvate still repressed histone gene expression in PDHA-1 siRNA treated cells. If pyruvate regulates histone gene expression via its conversion to acetyl-CoA, the global levels of H3K9ac should be increased and histone proteins will be increased instead of reduced. All these data indicate that pyruvate represses histone gene expression independent of LDH- and PDH-mediated pyruvate metabolism.

Histone gene expression is linked to cell cycle progression (44); however, we do not think pyruvate treatment inhibits histone gene expression by causing cell cycle arrest based on the following findings. The time-course experiment showed that pyruvate reduced histone gene expression as early as 0.5 h, suggesting that pyruvate represses histone genes prior to cell cycle arrest. By individually applying HU, thymidine and nocodazole to synchronize cells at G1/S, early S and G2/M, respectively, we observed that pyruvate treatment significantly reduces histone gene expression in all these synchronized cells. Moreover, pyruvate induces the expression of NAMPT in these synchronized cells, supporting that pyruvate represses histone gene expression independent of cell cycle progression. We also knocked down the expression of Cyclin A2 to cause cells to arrest at the G2/M phase and found that pyruvate represses histone gene expression in Cyclin A2 knockdown cells. In addition, overexpression of histones H3 and H4 partly alleviates the cell cycle arrest by pyruvate treatment.

NAMPT is a rate-limiting enzyme in the NAD^+^ salvage pathway and plays an important role in many cellular processes (56). NAMPT is a potent oncogene and highly expressed in cancer cells and cancer tissues such as colon cancer, glioblastoma, etc (57,58). NAMPT is also involved in oxidative stress response as knockdown NAMPT sensitizes prostate cancer cells to H_2_O_2_ by regulating the expression of anti-oxidant genes catalase (CAT) and manganese superoxide dismutase (SOD) (59). Here, we identified an anti-tumorigenic function of NAMPT in the presence of exogenous pyruvate: NAMPT is required for pyruvate to repress histone gene expression. NAMPT was not detected at histone gene promoters and no direct interaction was observed between NAMPT and SIRT1 (data not shown), which is consistent with the reported results (27). As NAD^+^ has been shown to restrict the diffusion of SIRT1 in specific subnuclear territories (60), the function of NAMPT in SIRT1 activation and histone gene expression could be primarily mediated by NAD^+^.

SIRT1 is an extensively-studied NAD^+^-dependent histone deacetylase, which regulates many biological processes, including regulation of gene expression, cellular metabolism, stress response, aging, autophagy and chemo-resistance; however, its role in tumorigenesis is ambiguous (61). SIRT1 expression is significantly elevated in many solid tumors, such as prostate cancer (62). SIRT1 has been shown to silence tumor suppressors, e.g., P53, or activate tumor drivers, e.g., the PTEN/PI3K/AKT pathway, thereby promoting tumorigenesis (63). However, numerous studies reported the role of SIRT1 as a tumor suppressor (64). For example, SIRT1 transgenic or overexpression mice are less susceptible to cancers, i.e. liver carcinogenesis, lung adenocarcinomas and intestinal cancers (64,65). Here, our data show that although knockdown of SIRT1 has no significant effect on cell growth, SIRT1 is required for pyruvate to inhibit cancer cell proliferation. Overexpression of SIRT1 can enhance the inhibitory effect of pyruvate on cell growth. Therefore, the precise tumorigenic and anti-tumorigenic functions of SIRT1 are highly dependent on the genetic context of the cell or tumor in question. Identifying the precise function of SIRT1 in tumorigenesis helps to develop anti-cancer therapy. For example, the well-known SIRT1 activator, resveratrol is a natural compound with potential therapeutic use in the treatment of many diseases, including cancer, diabetes, and other metabolic disorders (66). In this study, we found that resveratrol inhibits cancer cell proliferation by activating SIRT1 to repress histone gene expression. Given their similar functions in repressing histone gene expression, resveratrol and pyruvate have a synergistic inhibitory effect on tumor cell growth.

Collectively, we find that pyruvate inhibits cell proliferation and tumor growth primarily by acting as a signaling molecule to repress histone gene expression. Moreover, we uncover the mechanism by which pyruvate represses histone gene expression via the NAMPT–NAD^+^–SIRT1 pathway.

## DATA AVAILABILITY

The GEO accession number for the raw RNA-seq dataset in this paper is GEO: GSE135831.

## Supplementary Material

gkz864_Supplemental_File
