# Supplementary material for: Exogenous pyruvate represses histone gene expression and inhibits cancer cell proliferation via the NAMPT–NAD+–SIRT1 pathway
Source: Nucleic Acids Res. 2019 Oct 10;47(21):11132–50. doi: 10.1093/nar/gkz864 (PMC6868375; doi:10.1093/nar/gkz864)
Supplement: gkz864_Supplemental_File [file gkz864_supplemental_file.pdf]

Supplementary_Fig_S1

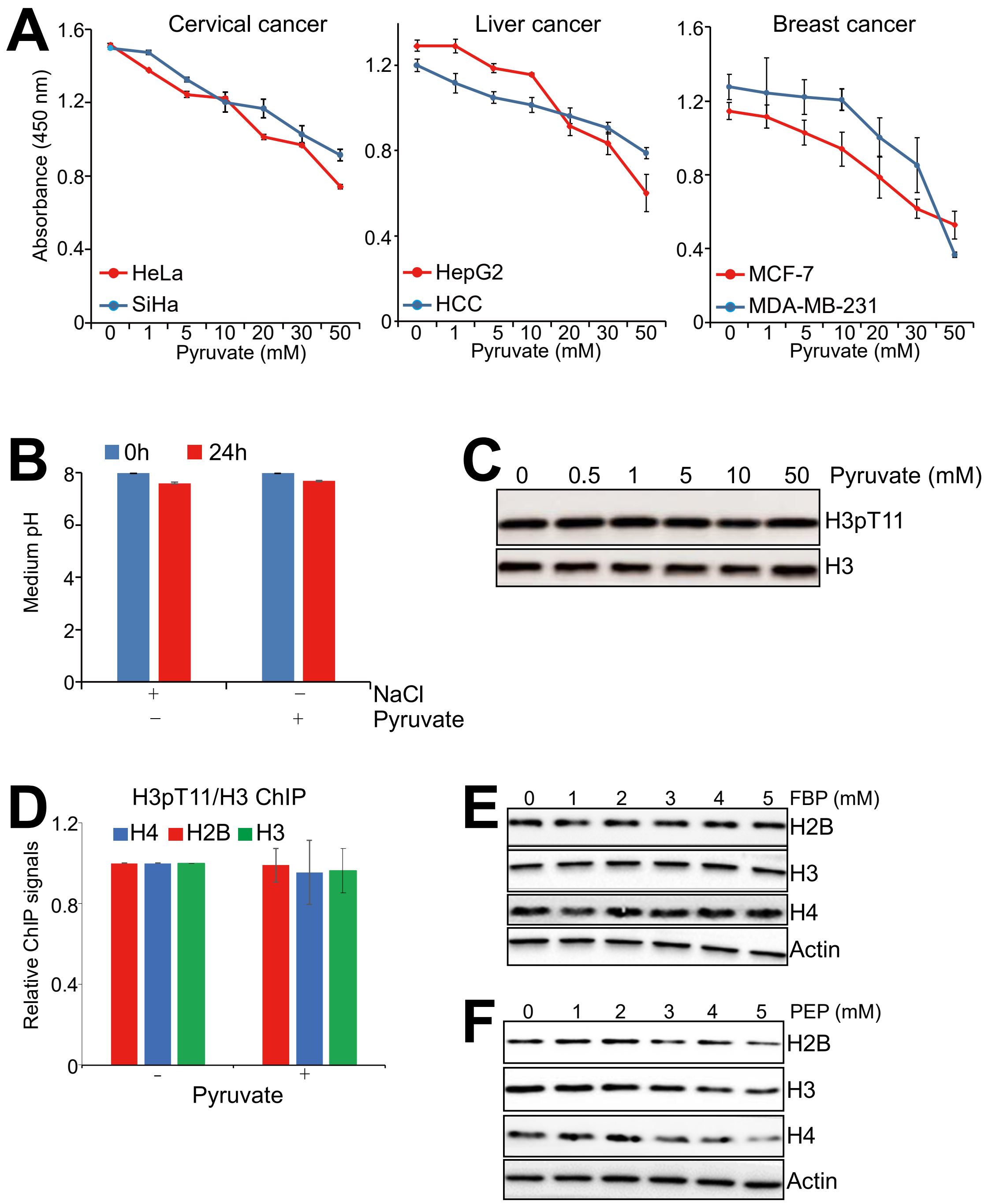

Figure S1. (A) Effect of pyruvate on the proliferation of cervical cancer cells (HeLa, SiHa), liver cancer cells (HepG2, HCC), and breast cancer cells (MCF-7, MDA-MB-231). Cells were treated with different concentrations of sodium pyruvate for 24 h and cell growth was determined by CCK-8 kit. Results are means ± SE of five independent experiments. (B) Pyruvate does not change the pH of the cell culture media. HeLa cells were treated with 5 mM sodium pyruvate for 24 h. As a control, HeLa cells were treated with 5 mM NaCl for 24 h. The pH of the media was determined. (C) Effect of pyruvate on H3T11 phosphorylation (H3pT11) in HeLa cells as determined by Western blots. (D) Effect of pyruvate on H3pT11 at histone genes by ChIP. HeLa cells were treated with 5 mM sodium pyruvate for 24 h. Results are means ± SE of three independent experiments. (E and F) Effect of 0-5 mM FBP (E) and PEP (F) on histone protein levels in HeLa cells.

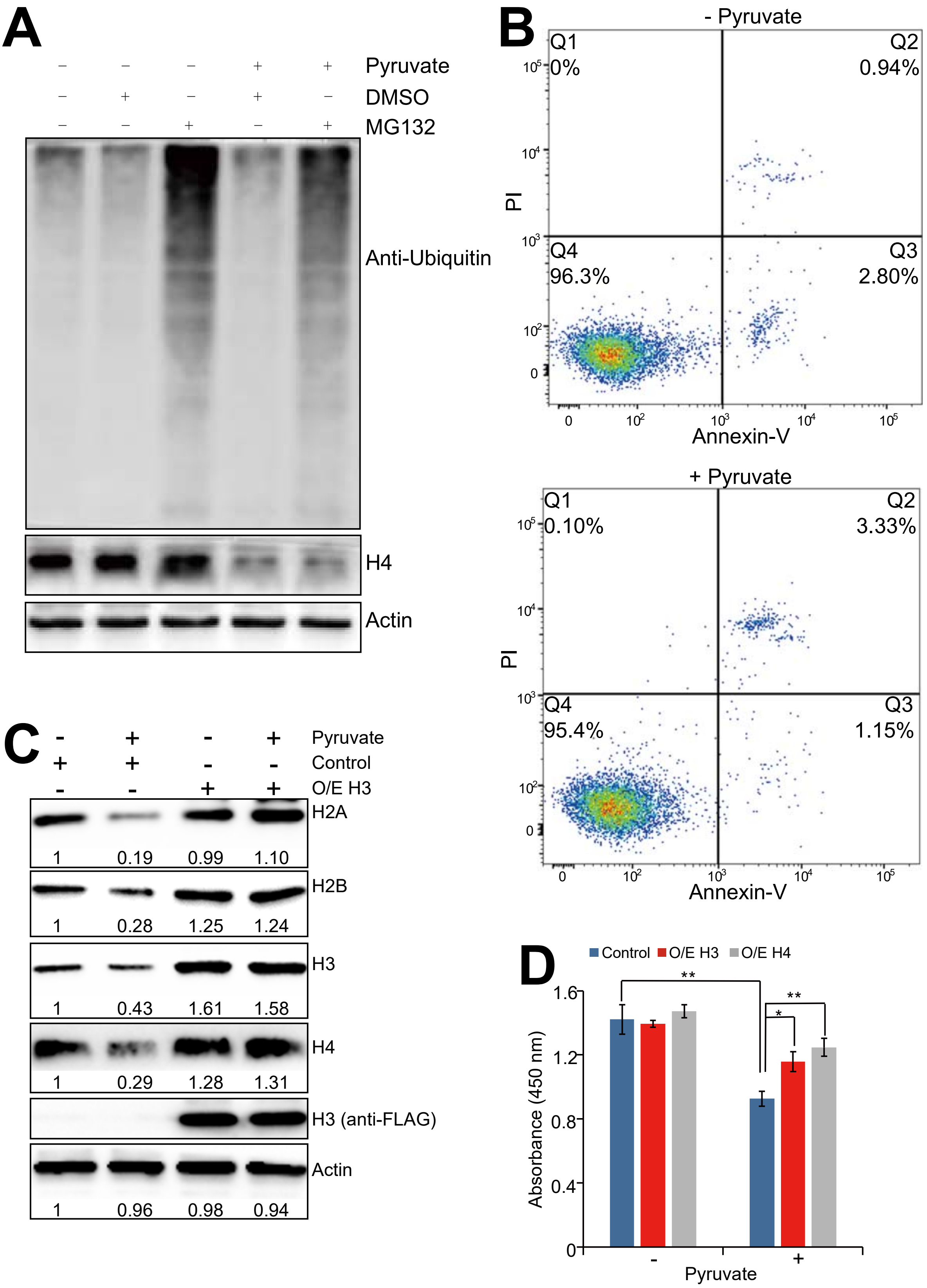
A
Pyruvate
DMSO
MG132
Anti-Ubiquitin
H4
Actin
B
- Pyruvate
Q1
0%
Q2
0.94%
Q4
96.3%
Q3
2.80%
PI
Annexin-V
+ Pyruvate
Q1
0.10%
Q2
3.33%
Q4
95.4%
Q3
1.15%
PI
Annexin-V
C
Pyruvate
Control
O/E H3
H2A
1 0.19 0.99 1.10
H2B
1 0.28 1.25 1.24
H3
1 0.43 1.61 1.58
H4
1 0.29 1.28 1.31
H3 (anti-FLAG)
Actin
1 0.96 0.98 0.94
D
Control
O/E H3
O/E H4
Absorbance (450 nm)
Pyruvate

Figure S2. (A) Effect of pyruvate on histone stability. HeLa cells were treated with or without pyruvate along with DMSO or MG132. After 24 h, cells were harvested and analyzed by Western blots with indicated antibodies. (B) Effect of pyruvate on cell apoptosis. HeLa cells were treated with 5 mM NaCl or sodium pyruvate for 24 h. Cells were stained using Annexin V-EGFP/PI and analyzed by flow cytometry. (C and D) Overexpression of histone H3 (O/E H3) or H4 (O/E H4) partly rescued the inhibitory effect of pyruvate on HeLa cell growth. *, $P<0.05$; **, $P<0.01$.

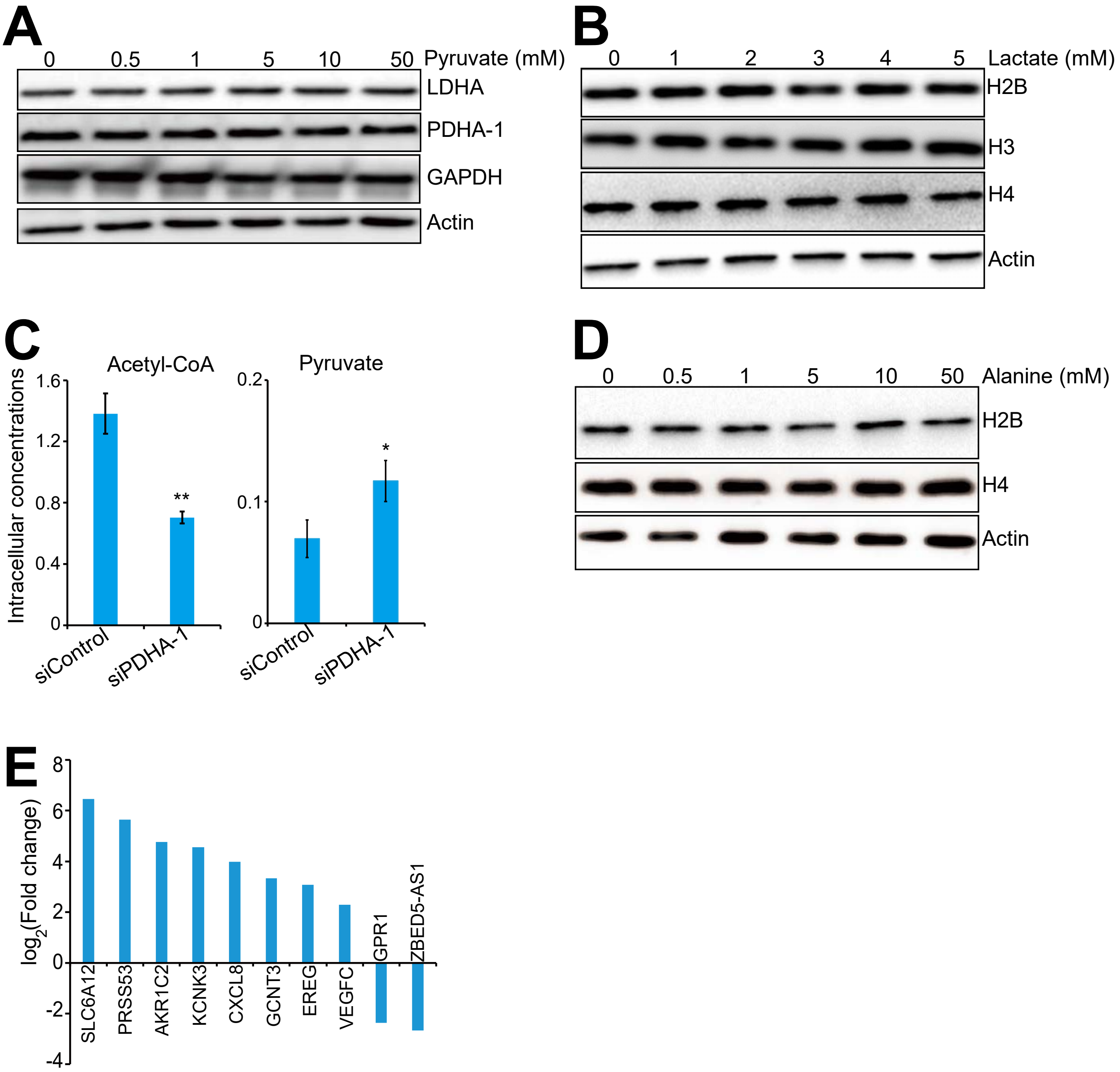

Figure S3. (A) Effect of pyruvate on protein levels of LDHA and PDHA-1 in HeLa cells. (B) Effect of lactate on histone proteins in HeLa cells. (C) Measurement of acetyl-CoA and pyruvate levels in siControl and siPDHA-1 transfected HeLa cells. Cells were not treated with sodium pyruvate. (D) Effect of alanine on histone proteins in HeLa cells. (E) Effect of pyruvate on transcription of selected genes. Data were extracted from our RNA-seq data.

Supplementary_Fig_S4

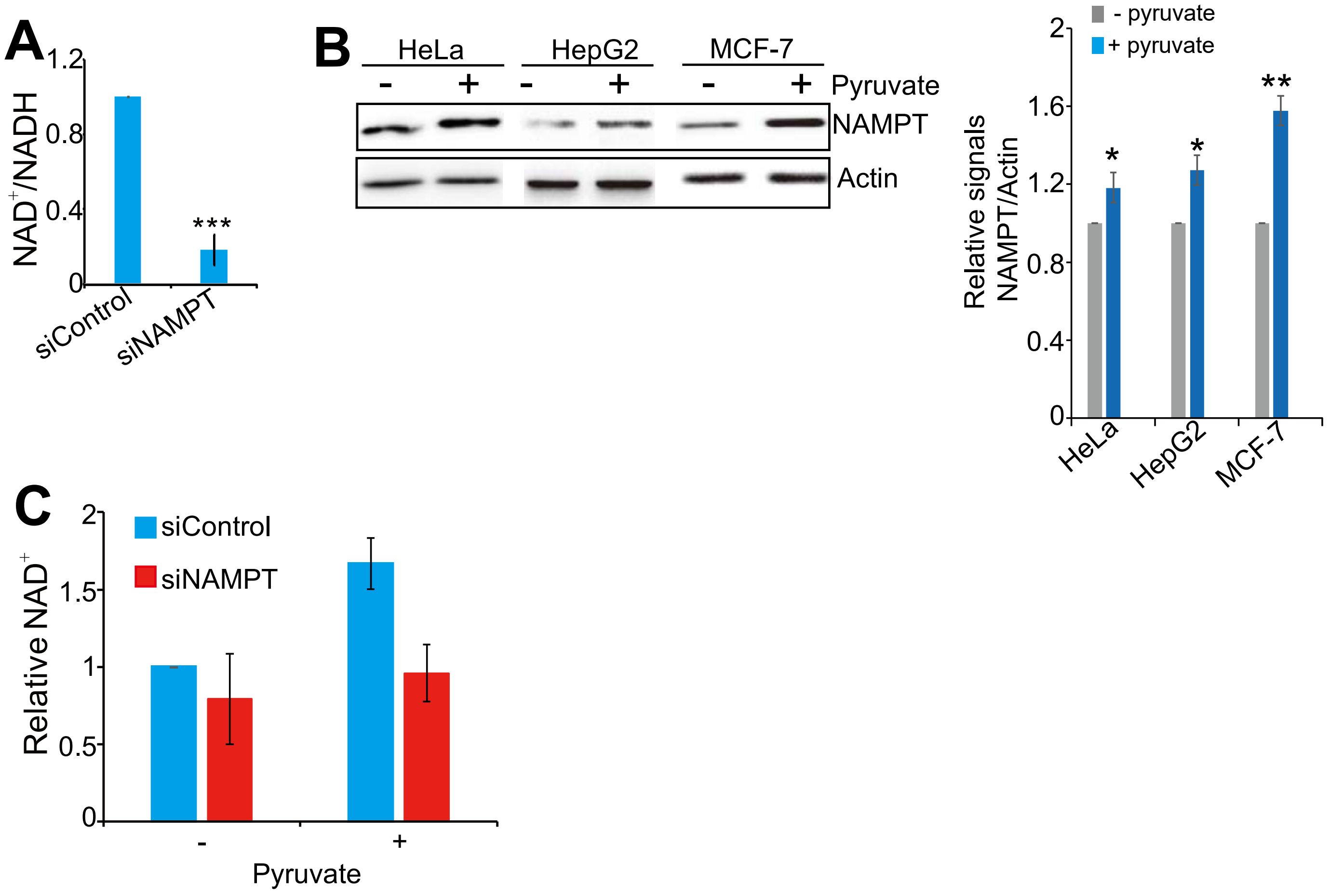

Figure S4. Pyruvate represses histone gene expression by inducing the expression of NAMPT and increasing the ratio of NAD^+/NADH. (A) Effect of NAMPT on intracellular NAD^+/NADH ratio. The ratio of NAD^+/NADH was determined in scrambled siRNA (siControl) and NAMPT siRNA (siNAMPT) treated HeLa cells. Data represent means ± SE (n=3). ***, P<0.001. (B) Effects of pyruvate on NAMPT expression in HeLa, HepG2 and MCF-7 cells as determined by Western blots. The relative intensities of NAMPT/Actin were quantified using Image J (n=3). *, P<0.05; **, P<0.01. (C) NAMPT is required for pyruvate to increase NAD^+.

Supplementary_Fig_S5

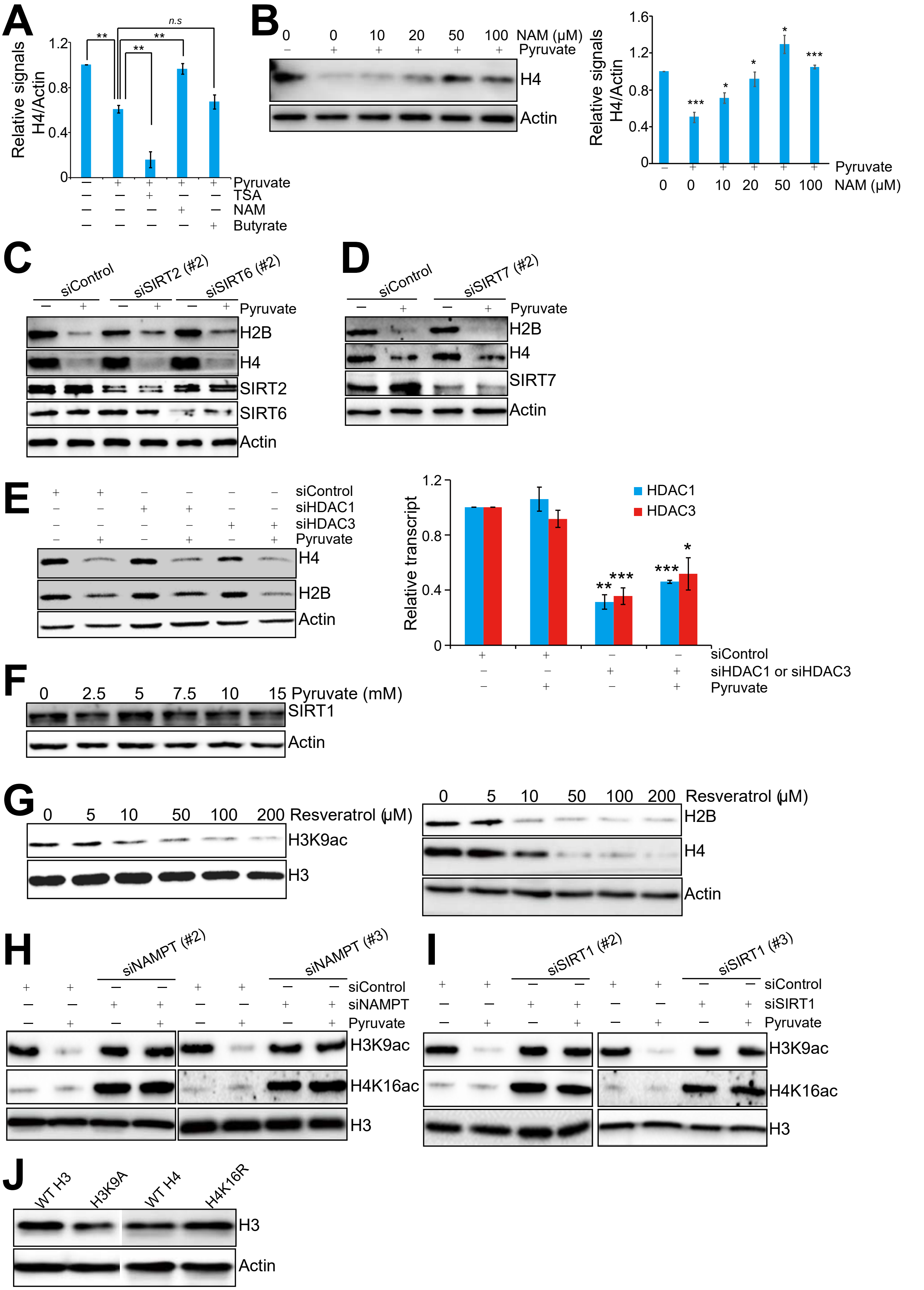

Figure S5. (A) Quantitation of Western blots data in Figure 4A by Image J. **, P<0.01; *n.s*, no significance. (B) Effect of pyruvate and various concentrations of nicotinamide (NAM) on histone gene expression. Data represent means ± SE (n=3). *, P<0.05; ***, P<0.001. (C-D) Effect of pyruvate on histone proteins in siControl, siSIRT2 (#2), siSIRT6 (#2), and siSIRT7 (#2) transfected HeLa cells as determined by Western blots. (E) Effect of pyruvate on histone proteins in siControl, siHDAC1, and siHDAC3 transfected HeLa cells as determined by Western blots. Right panel: Analysis of the knockdown efficiency by qRT-PCR. Data represent means ± SE (n=3). *, P<0.05; **, P<0.01; ***, P<0.001. (F) Effect of pyruvate on SIRT1 protein levels in HeLa cells as determined by Western blots. (G) Effect of resveratrol on H3K9ac and histone proteins in HeLa cells as determined by Western blots. Data displayed are typical of three independent replicates. (H) Effect of pyruvate on H3K9ac and H4K16ac in siControl, siNAMPT (#2) and siNAMPT (#3) transfected HeLa cells. (I) Effect of pyruvate on H3K9ac and H4K16ac in siControl, siSIRT1 (#2) and siSIRT1 (#3) transfected HeLa cells. (J) Effect of H3K9A and H4K16R mutations on histone protein levels in budding yeast.

Supplementary_Fig_S6

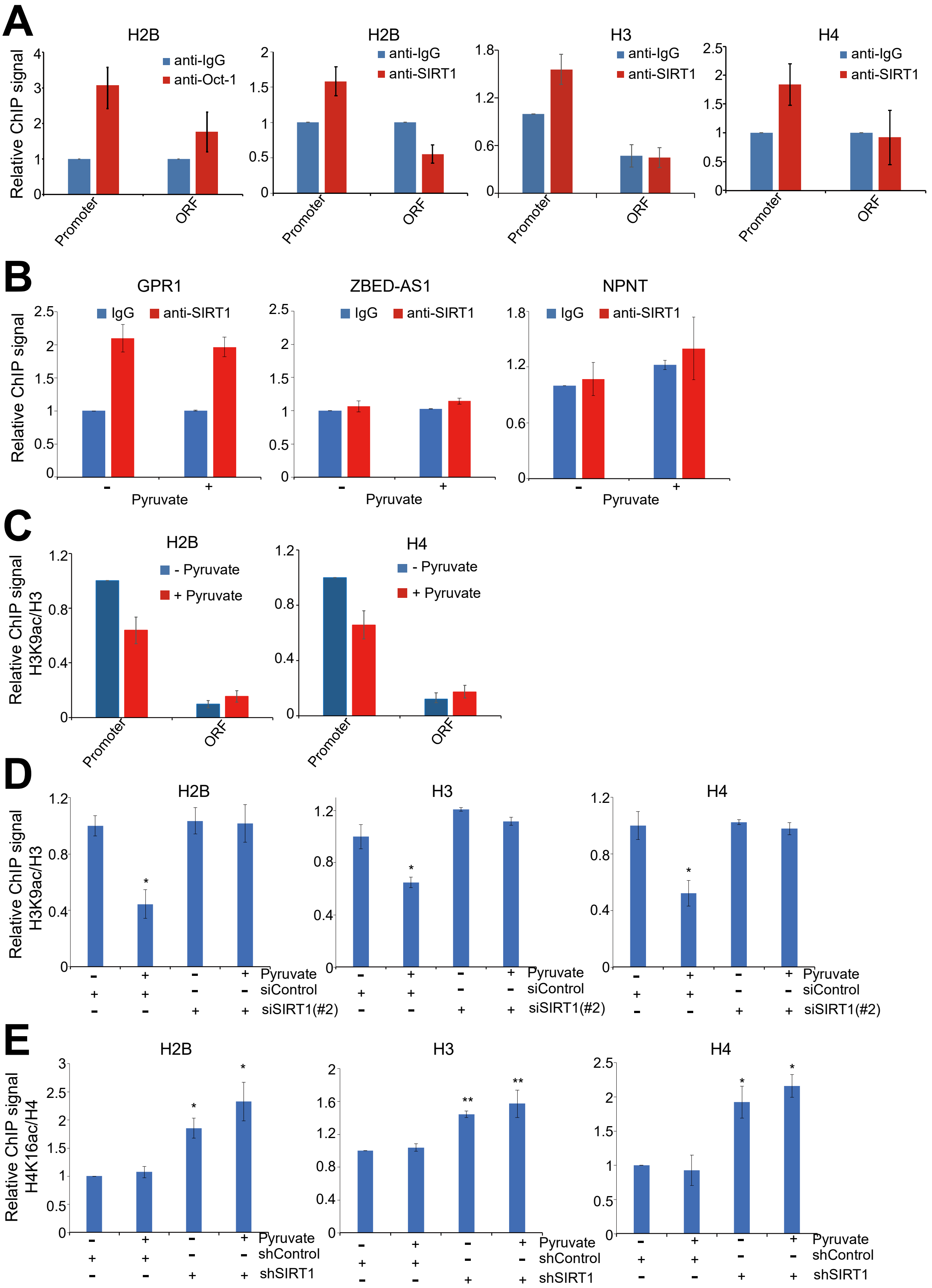

Figure S6. Pyruvate promotes the binding of SIRT1 at histone gene promoters to reduce H3K9ac. (A) ChIP analysis of SIRT1 occupancy at H2B, H3 and H4 promoters and coding regions in HeLa cells. Oct-1 is a H2B transcription activator and used as a positive control. (B) ChIP analysis of the effect of pyruvate on SIRT1 binding at GPR1, ZBED-AS1 and NPNT promoter regions in HeLa cells. IgG was used as negative controls. Results are means ± SE (n=3). (C) ChIP analysis of the effect of pyruvate on the ratio of H3K9ac/H3 at histone genes in HeLa cells. Results are means ± SE (n=3). (D) ChIP analysis of the effect of pyruvate on the ratio of H3K9ac/H3 at histones H2B, H3 and H4 promoters in siControl and siSIRT1 (#2) HeLa cells. Results are means ± SE (n=3). *, $P<0.05$. (E) ChIP analysis of the effect of pyruvate on the ratio of H4K16ac/H4 at histone H2B, H3 and H4 promoters in shControl and shSIRT1 HeLa cells. Results are means ± SE (n=3). *, $P<0.05$; **, $P<0.01$.

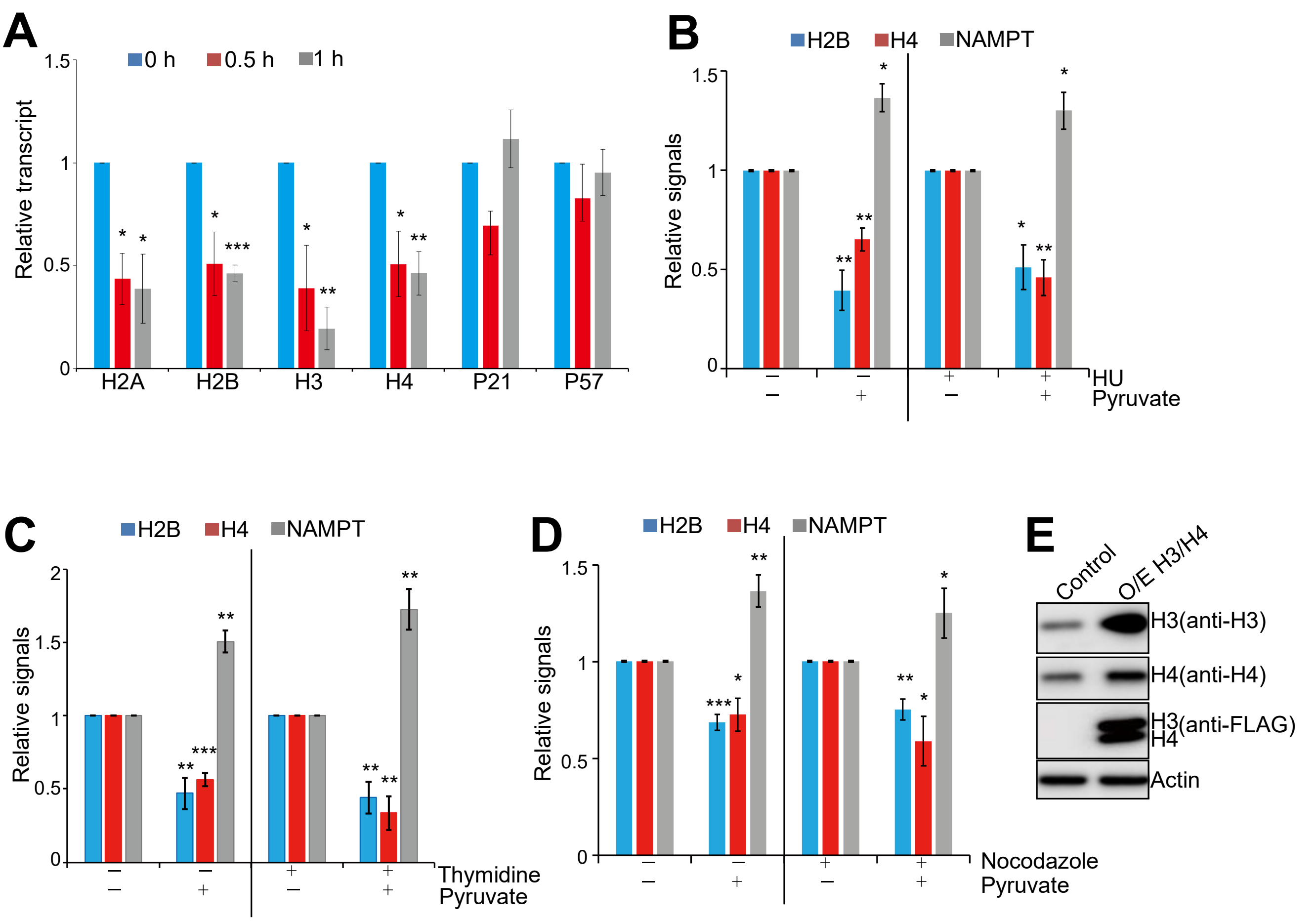

Figure S7. (A) qRT-PCR analysis of the effect of pyruvate on transcription of histone genes, P21 and P57. HeLa cells were treated with 5 mM pyruvate for 0, 0.5 h and 1 h. Data represent means ± SE (n=3). *, P<0.05; **, P<0.01; ***, P<0.001. (B-D) Pyruvate significantly represses the expression of histone genes but increases the expression of NAMPT in HU- (B), thymidine- (C) and nocodazole- (D) synchronized HeLa cells. Asynchronized HeLa cells were used as a control. Shown are the relative intensities of H2B/Actin, H4/Actin and NAMPT/Actin in Figure 5D-F. Data represent means ± SE (n=3). *, P<0.05; **, P<0.01; ***, P<0.001. (E) Western blots analysis of histones in HeLa cells transfected with control pCMV (Control) and pCMV-H3/H4 (O/E H3/H4) plasmids in Figure 5H.

Supplementary_Fig_S8

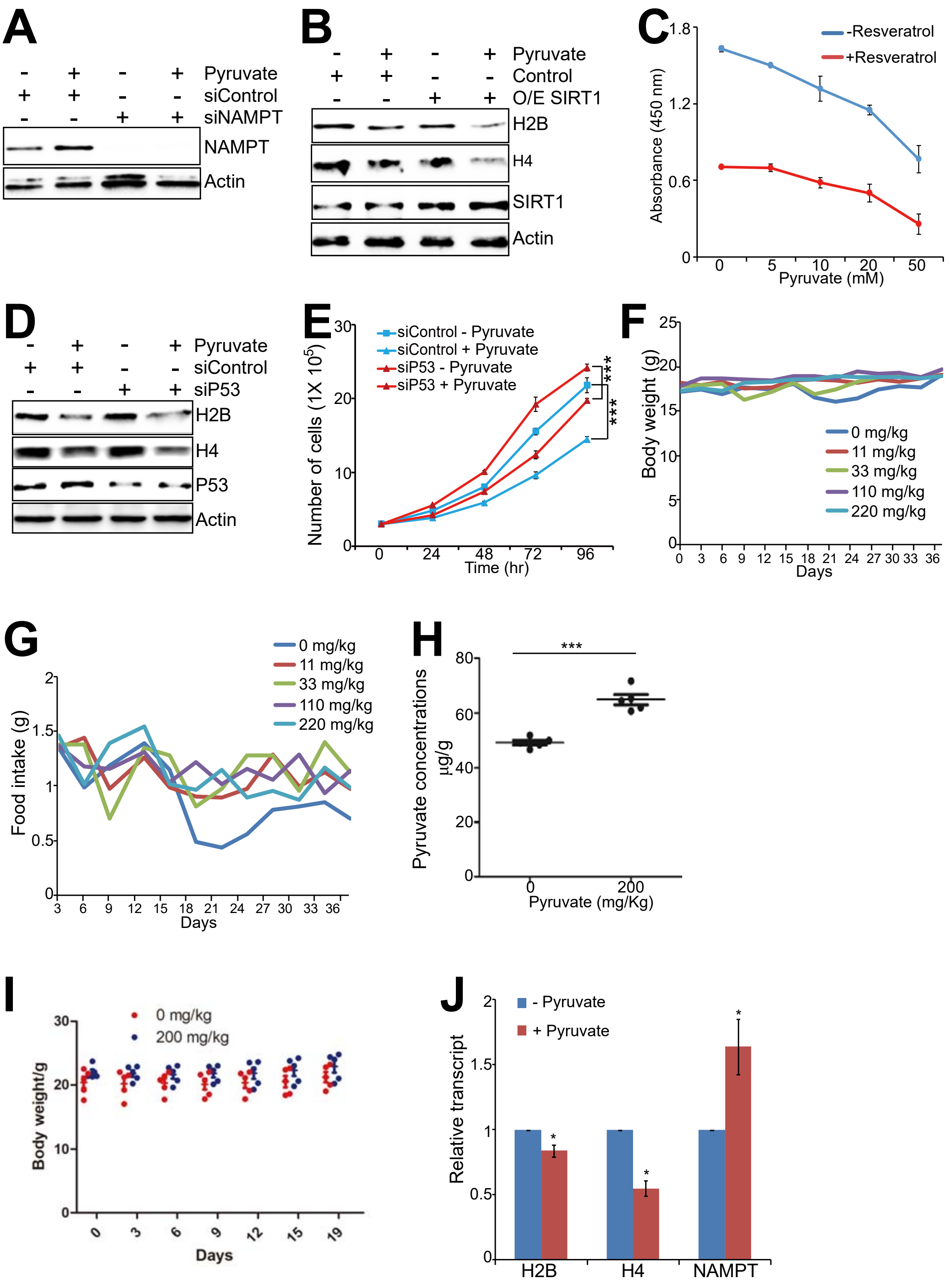

Figure S8. (A) Western blots analysis of NAMPT expression in siControl and siNAMPT transfected HeLa cells. (B) Effect of pyruvate on histone proteins in HeLa cells transfected with pCMV (Control) and pCMV-SIRT1 (O/E SIRT1). (C) Pyruvate and resveratrol synergistically inhibit the proliferation of HeLa cells. Cells were treated with or without 100 μM resveratrol along with 0-50 mM sodium pyruvate. (D and E) Effect of pyruvate on histone gene expression (D) and cell proliferation (E) in siControl and siP53 transfected HeLa cells. ***, P<0.001. (F and G) Effect of pyruvate administration on body weight (F) and food intake (G) of normal mice. The mice (n=6) were administered with different amounts of pyruvate and their body weight and food intake were measured at the indicated days. (H) Pyruvate administration significantly increased the pyruvate levels in the kidneys of xenografted mice. Data represent means ± SE (n=5). ***, P<0.001. (I) Effect of pyruvate treatment on body weight of xenografted mice. No significant difference was observed between PBS and pyruvate treated groups (five mice per group). (J) qRT-PCR analysis of histone genes and NAMPT transcription in xenograft tumors administered with PBS or pyruvate. Data represent means ± SE (n=3). *, P<0.05.

Supplementary_Fig_S9

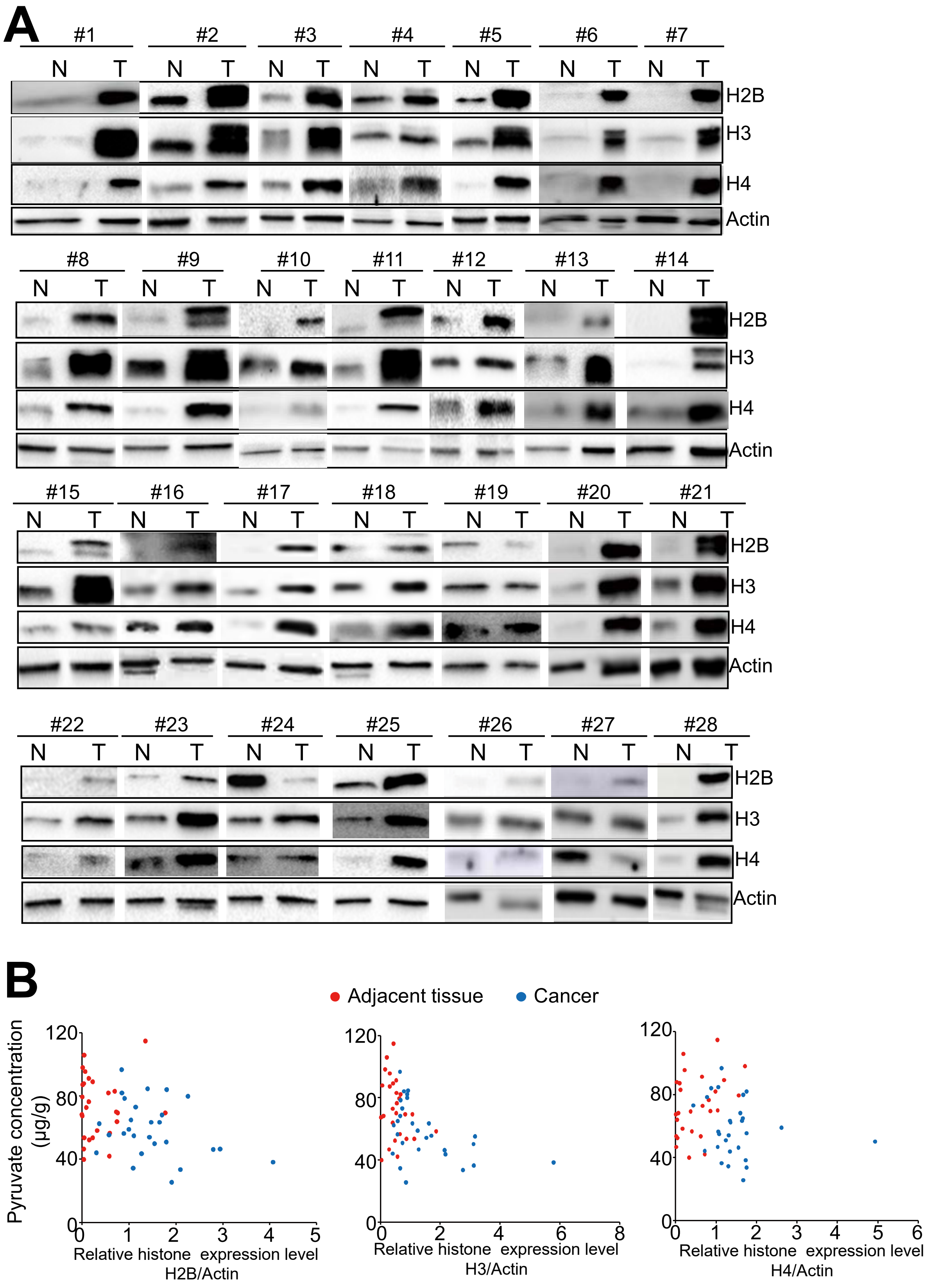

Figure S9. Histone genes are up-regulated in cancer. (A) Western blots analysis of histone proteins in 28 paired tissue specimens of cervical cancer (T) and adjacent normal tissues (N). 5 pairs of samples have been shown in Figure 7C. (B) Scatter plots for pyruvate concentrations and histone protein levels in 28 paired tissue specimens. The Western blots data of histone proteins in 28 paired tissue specimens in Figure S9A were quantitated by Image J.

Supplementary_Fig_S10

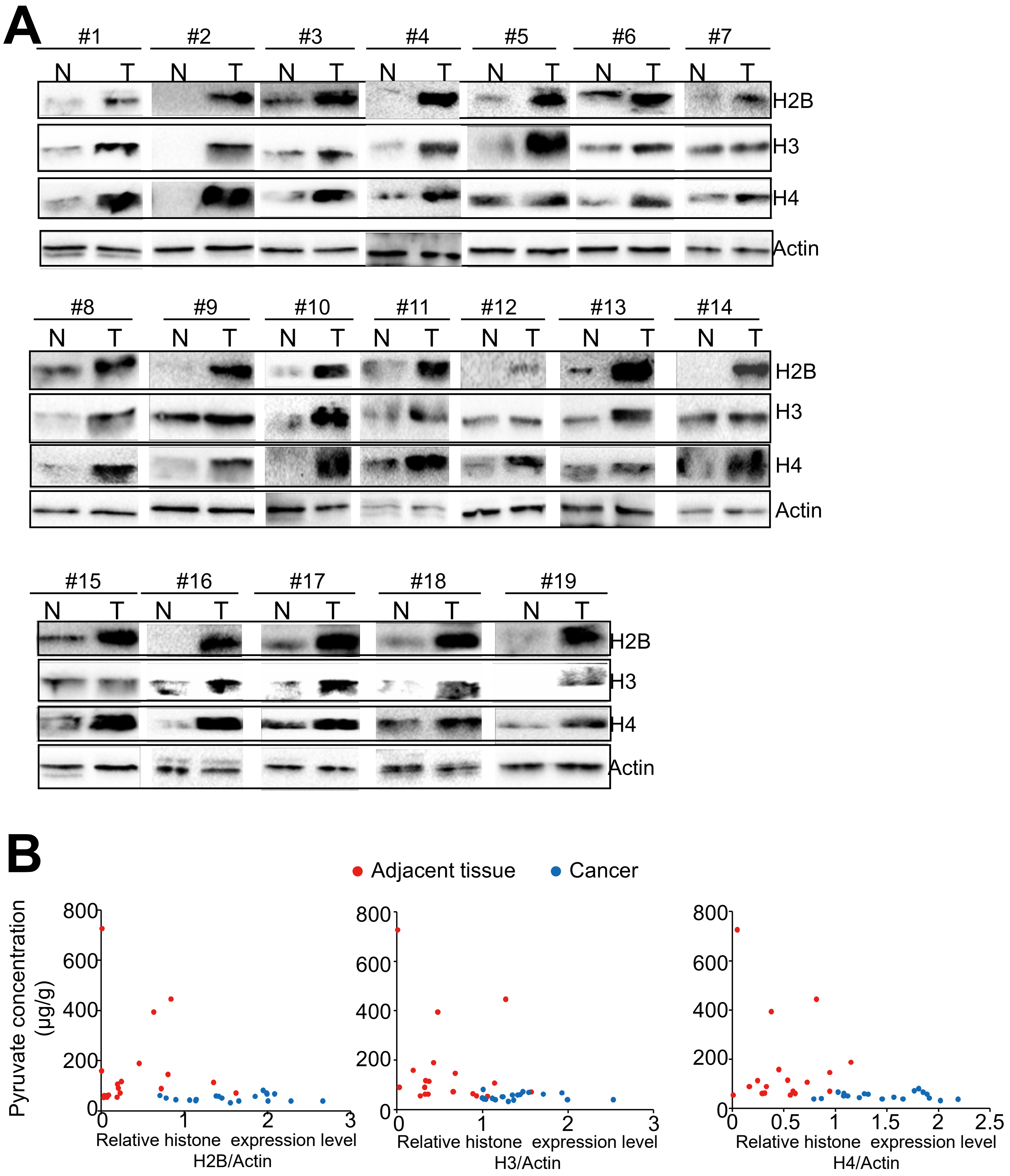

Figure S10. Histone genes are up-regulated in cancer. (A) Western blots analysis of histone proteins in 19 paired tissue specimens of lung cancer (T) and adjacent normal tissues (N). 5 pairs of samples have been shown in Figure 7D. (B) Scatter plots for pyruvate concentrations and histone protein levels in 19 paired tissue specimens. The Western blots data of histone proteins in 19 paired tissue specimens in Figure S10A were quantitated by Image J.

Supplementary_Fig_S11

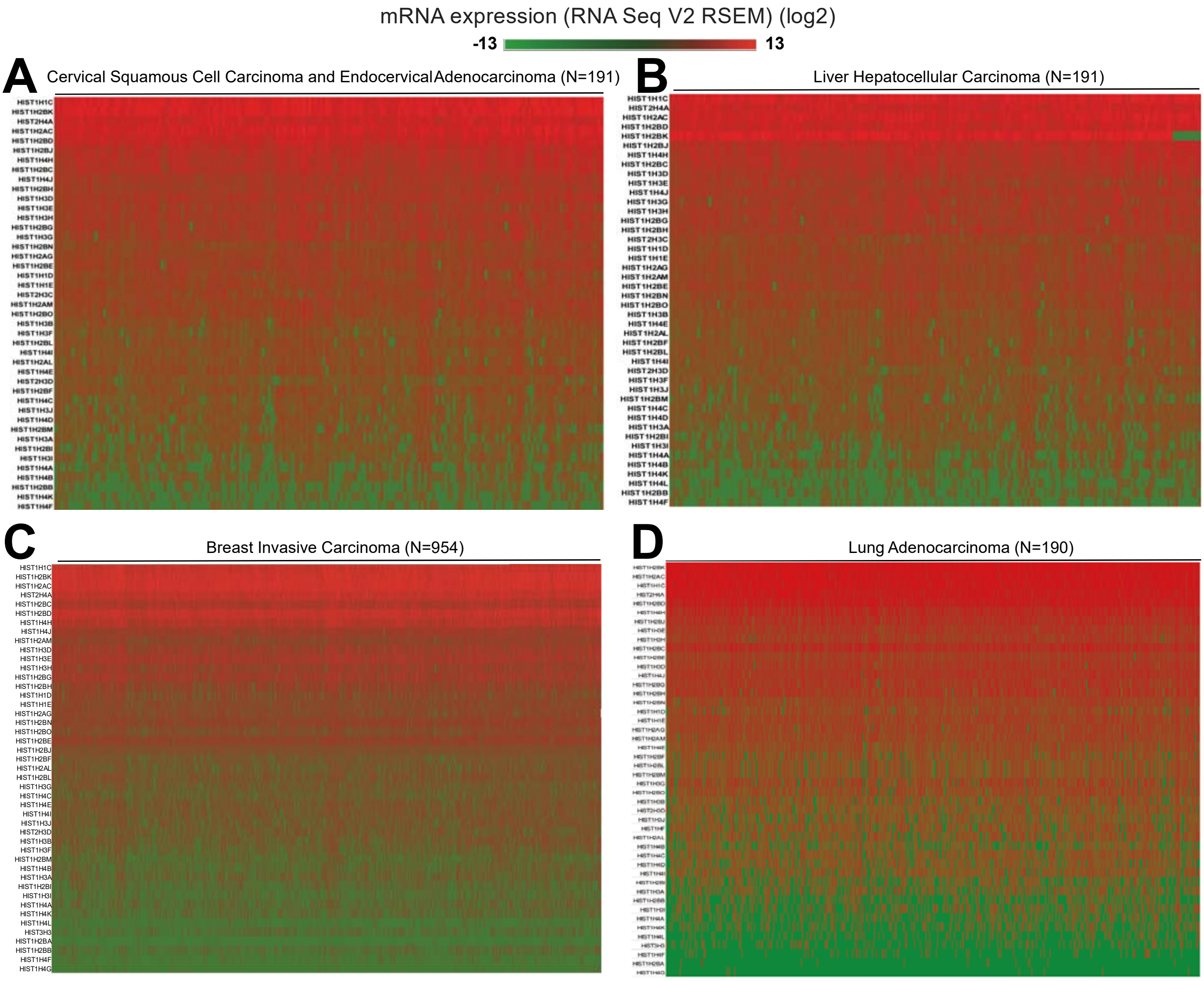

Figure S11. The heatmap shown histone gene expression in 191 cervical squamous cell carcinoma and endocervical adenocarcinoma (A), 191 liver hepatocellular carcinoma (B), 954 breast invasive carcinoma (C), and 190 lung adenocarcinoma (D).

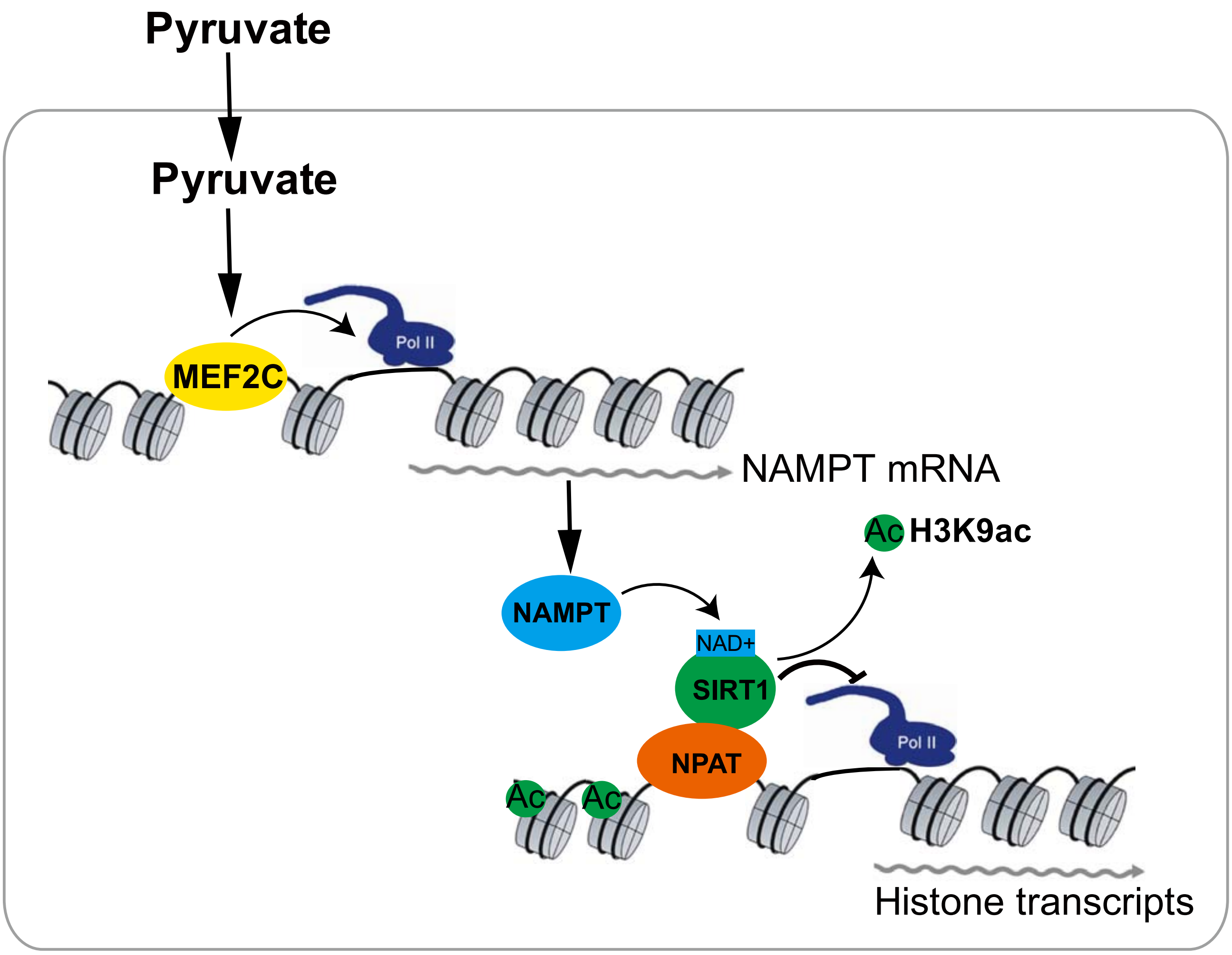

Figure S12. Proposed model for histone gene repression by exogenous pyruvate. Pyruvate treatment upregulates the expression of NAMPT via MEF2C, which increases the intracellular NAD^+. Elevated NAD^+ then enhances the binding and activity of SIRT1 in a NPAT-dependent manner at histone genes to deacetylate histones, i.e. H3K9 and repress histone gene transcription.

Table S1 List of siRNA used in this study

Gene name	Sequence
scrambled siRNA	5’-TTCTCCGAACGTGTCACGT-3’
si-NAMPT#1	5’-GGUAAGAAGUUUCCUGUUATT-3’
	5’-UAACAGGAAACUUCUUACCTT-3’
si-NAMPT#2	5’-CCUACAAGGUUACUCACUAUATT-3’
	5’-UAUAGUGAGUAACCUUGUAGGTT-3’
si-NAMPT#3	5’-GUAACUUAGAUGGUCUGGAAUTT-3’
	5’-AUUCCAGACCAUCUAAGUUACTT-3’
si-SIRT1#1	5’-GCUUGAUGGUAAUCAGUAUCUTT-3’
	5’-AGAUACUGAUUACCAUCUUGCTT-3’
si-SIRT1#2	5'-GCGGGAAUCCAAAGGAUAAUUTT-3'
	5'-AAUUAUCCUUUGGAUUCCCGCTT-3'
si-SIRT1#3	5'-GCUUGAUGGUAAUCAGUAUCUTT-3'
	5'-AGAUACUGAUUACCAUCAAGCTT-3'
si-LDHA#1	5’-GGAGAAAGCCGUCUUAAUUTT-3’
	5’-AAUUAAGACGGCUUUCUCCTT-3’
si-LDHA#2	5’-GGCAAAGACUAUAAUGUAATT-3’
	5’-UUACAUUAUAGUCUUUGCCTT-3’
si-LDHA#3	5’-AAAGUCUUCUGAUGUCAUATT-3’
	5’-UAUGACAUCAGAAGACUUUTT-3’
si-PDHA#1	5’-GCUGGUAGCAUCCCGUAAUUTT-3’
	5’-AAUUACGGGAUGCUACCAGCTT-3’
si-PDHA#2	5’-UCCAAUCAGUGGAUCAAGUTT-3’
	5’-ACUUGAUCCACUGAUUGGATT-3’
si-PDHA#3	5’-CUAAUGGAGUUGAAAGCAGATT-3’
	5’-UCUGCUUUCAACUCCAUUAGTT-3’
si-HDAC1	5’-CCCGGAGGAAAGUCUGUUATT-3’
	5’-UAACAGACUUUCCUCCGGGTT-3’
si-HDAC3	5’-GAUGCUGAACCAUGCACCUTT-3’
	5’-AGGUGCAUGGUUCAGCAUCTT-3’
si-SIRT2#1	5’-CGGUACAUGCAGAGCGAACTT-3’
	5’-GUUCGCUCUGCAUGUACCGTT-3’
si-SIRT2#2	5’-GCCUCUAUGACAACCUAGATT-3’
	5’-UCUAGGUUGUCAUAGAGGCTT-3’
si-SIRT6#1	5’-UUCACUCCUGUUUAAGCUGTT-3’
	5’-CAGCUUAAACAGGAGUGAATT-3’

si-SIRT6#2	5’-GAAUGUGCCAAGUGUAAGATT-3’
	5’-UCUUACACUUGGCACAUUCTT-3’
si-SIRT7#1	5’-CACCUUUCUGUGAGAACGGAATT-3’
	5’-UUCCGUUCTCACAGAAAGGUGTT-3’
si-SIRT7#2	5’-GCCUGAAGGUUCUAAAGAATT-3’
	5’-UUCUUUAGAACCUUCAGGCTT-3’
si-Cyclin A2	5’-UAAACCUAAAGUGGGUUACAUGTT-3’
	5’-CAUGUAACCCACUUUAGGUUUATT-3’
si-P53	5’-CACCUCACUGCAUGGACGAUCUGTT-3’
	5’-CAGAUCGUCCAUGCAGUGAGGUGTT-3’
si-NPAT	5’-GGGUUUGCGAAGUGAGAAATT-3’
	5’-UUUCUCACUUCGCAAACCCTT-3’
si-MPC1	5’- CUAUGUCCGAAGCAAGGAUTT -3’
	5’- AUCCUUGCUUCGGACAUAGTT -3’
si-MCT1	5’- GAAACGAUCAGUCUUCCAATT-3’
	5’- UUGGAAGACUGAUCGUUUCTT -3’

Table S2 List of oligonucleotides used in this study

**Gene name**	**Sequence**
ChIP	
H2A-promoter	CTCTTCTGGGGTCCGCTTCTC
	GCGTTCAGGCTGGGATTTTAA
H2A-ORF	ACCATGTCTGGACGTGGCAA
	ATCTCGGCAGTTAGGTACTC
H3-promoter	GGCCTGTCTTCTTGTGAATTTAAAA
	GGAGCAGGAGGTGGACTTGG
H3-ORF	ATGGCTCGCACTAAGCAAACT
	CGCACAGGTTAGTGTCCTCAAAT
H4 -promoter	CACGGAAACAAGAAGCTCGCT
	GCAGCGCCTTTATACGACAGTT
H4-ORF	GCTAAGCGCCACCGTAAAGTA
	TGGCGTGCTCTGTATAGGTCA
H2B-promoter	ACACGGACCAATGGAAATGA
	CGGTTCAGGCATGGTAAGAC
H2B -ORF	CAGTGCTATGCCAGAGCCAGCGAA
	CTGTTTACTTAGCGCTGGTGTACTTGGTGA
SLC6A12-promoter	GTCGATAGTTACCCCTTCCCATA
	TACGCGCCACCCTGTCACGA
PRSS53-promoter	TCGCCCCCTTTCCCCTCCCA
	CGAGGAGGCGGGCGGGGACG
AKR1C2-promoter	GACCACTAGACCGACAAAAAG
	ATGTCCTGTCCCCACTTAGTC
VEGFC-promoter	CCCCTCTCTCCCTTGGTCTGT
	TCTGGCCCTTCTCTTCACCTG
CXCL8-promoter	TAAAAAGCCACCGGAGCACTC
	CGGTGGTTTCTTCCTGGCTCT
GCNT3-promoter	TTTGTTAAGAGCCCACTACCTAGAA
	GGCTGGAGCTAGGGTTTGAAA
EREG-promoter	ACTCTTGACCTCACCCCGTTAG
	ACGTTGGCTGTGTCCCTCTGA
KCNK3-promoter	GCGGTGGGTGGTGCTGAAGGG
	GCCGCCGCTGCTGCTGCCCCG
NAMPT-promoter	ACCATCACTGCCCCCAGATAG
	ACCATCACTGCCCCCAGATAG
qRT-PCR	
β-actin	GCCGACAGGATGCAGAAGGAGATCA
	AAGCATTTGCGGTGGACGATGGA
NAMPT	ACTTCTGGTAACTTAGATGGTCTG
	GTCCTGCTACTGTATCTGTTCC

HDAC1	GCACCCGGAGGAAAGTCTGTT
	CAGCCTCGATCTCCCTTCCTC
HDAC3	TTTGAAAACCTGAAGATGCTGAAC
	CTTCCCATTACTTTTTCCCTTTT
MPC1	GTAGCCAACTGGGGTCTTCC
	AACAGAAGCCAGTTCCGAGG
H2A	ACCATGTCTGGACGTGGCAA
	ATCTCGGCAGTTAGGTACTC
H2B	CAGTGCTATGCCAGAGCCAGCGAA
	CTGTTTACTTAGCGCTGGTGTACTTGGTGA
H3	ATGGCTCGCACTAAGCAAACT
	CGCACAGGTTAGTGTCCTCAAAT
H4	GCTAAGCGCCACCGTAAAGTA
	TGGCGTGCTCTGTATAGGTCA
MCT1	ATTGGAGGTCTTGGGCTTGC
	CATTCCACAATGGTCACCAA
MEF2C	TGCTGGTCTCACCTGGTAAC
	CCTCCCATTCCTTGTCCTGG
P21	TTGTACCCTTGTGCCTCGCTCA
	AGATCAGCCGGCGTTTGGAGT
P57	AGATCAGCGCCTGAGAAGTCGTC
	GGCTCTAAATTGGCTCACCGCAG
H2AZ	CCTCACCGCAGAGGTACTTG
	GTTGCAAGTGACGAGGGGTA
H1.4	GTCGGGTTCCTTCAAACTCA
	CTTCTTCGCCTTCTTTGGG
H3.3	ATGGCTCGTACAAAGCAGAC
	GGTTTCTTCACCCCTCCAGT
NASP	GCGTCCCAAATTGCCTGTTT
	GCTTCACTATCCACATCCAGA
NPAT	TTGTTACCCTCGGACGTAGCC
	GGACAGTAAGCAGGCTGGAA
HIRA	CAGGAGGATGACGAGAAGGA
	ACTGTTTGACCACCGCACAC
ATXN10	CATTGCCTCACGGAATGAA
	TGAGGTTCTCCTCCAGTTCT
SOCS2	ATGACCCTGCGGTGCCT
	AAAGTTCCTTCTGGTGCCTCT
pCMV Vector construction	
pCMV-H2A	ATGACAAGCTTGCGGCCGCGATGTCTGGTCGTGGCAAACA
	TCAGATCTATCGATGAATTTTATTTGCTTTTGGCTTTGTGGCTT
pCMV-H2B	ATGACAAGCTTGCGGCCGCGATGCCTGAACCGGCAAAATC
	TCAGATCTATCGATGAATTTCACTTGGAGCTGGTGTACTTG

pCMV-MEF2C	ATGACAAGCTTGCGGCCGCGATGGGGAGAAAAAAGATTCAGA
	TCAGATCTATCGATGAATTTCATGTTGCCCATCCTTCA
pCMV-NAMPT	GACAAGCTTGCGGCCATGAATCCTGCGGCA
	CAGATCTATCGATGAATTCGCCTAATGATGTGCTGCTTCC
pCMV-SIRT1	ACGATGACAAGCTTGCGGCCATGGCGGACGAGGCGGCCCT
	CAGATCTATCGATGAATTCGCCTATGATTTGTTTGATGGATAGTTCA

Table S3 List of antibodies used in this study

Antibodies	SOURCE	IDENTIFIER
H4	Abcam	ab10158
H3	Abcam	ab1791
H2B	Cell signaling Technology	12364S
H2A	proteintech	10445-1-AP
H3pT11	Abcam	ab5168
PKM2	Cell signaling Technology	D78A4
NAMPT	proteintech	11776-1-AP
FLAG M2	Sigma	F1804-1MG
SIRT1	Cell signaling Technology	2310S
H3K9ac	Cell signaling Technology	9649S
H4K16ac	Merk Millipore	07-329
Oct-1	Cell signaling Technology	4428S
Ubiquitin (P4D1)	Cell signaling Technology	3936S
GAPDH	proteintech	10494-1-AP
PDHA-1	proteintech	18068-1-AP
LDHA	proteintech	19987-1-AP
Cyclin E1	Cell signaling Technology	4129T
Cyclin A	Santa Cruz Biotechnology	sc-271682
Cyclin B1	Abcam	ab32053
Cyclin A2	Wanleibio	WL02964
SIRT2	ZENBO	200474
SIRT6	ZENBO	200499-6C9
SIRT7	CUSABIO	CSB-PA885703LA01HU
Pan acetyl lysine	Cell signaling Technology	9441S
MEF2C	Santa Cruz Biotechnology	sc-365862
P53	Cell signaling Technology	2527T
